# Effective Enantiodiscrimination in Electroanalysis Based on a New Inherently Chiral 1,1′-Binaphthyl Selector Directly Synthesizable in Enantiopure Form

**DOI:** 10.3390/molecules25092175

**Published:** 2020-05-06

**Authors:** Giorgia Bonetti, Serena Arnaboldi, Sara Grecchi, Giulio Appoloni, Elisabetta Massolo, Sergio Rossi, Rocco Martinazzo, Francesco Orsini, Patrizia R. Mussini, Tiziana Benincori

**Affiliations:** 1 Dipartimento di Scienza ed Alta Tecnologia, Università degli Studi dell’Insubria, 22100 Como, Italy; gbonetti@uninsubria.it (G.B.); appogiulio@yahoo.it (G.A.); 2 Dipartimento di Chimica, Università degli Studi di Milano, 20133 Milano, Italy; serena.arnaboldii@unimi.it (S.A.); sara.grecchi@unimi.it (S.G.); elisabetta.massolo@unimi.it (E.M.); sergio.rossi@unimi.it (S.R.); rocco.martinazzo@unimi.it (R.M.); patrizia.mussini@unimi.it (P.R.M.); 3 Dipartimento di Fisica, Università degli Studi di Milano, 20133 Milano, Italy; francesco.orsini@unimi.it

**Keywords:** inherent chirality, 1,1’-binaphtyl monomer, electrodeposited films, chiral voltammetry, magnetoelectrochemistry, DOPA enantiodiscrimination, tryptophan enantiodiscrimination

## Abstract

Enantioselective electroanalysis, which aims to discriminate the enantiomers of electroactive chiral probes in terms of potential difference, is a very attractive goal. To achieve this, its implementation is being studied for various "inherently chiral" selectors, either at the electrode surface or in the medium, yielding outstanding performance. In this context, the new inherently chiral monomer Naph_2_T_4_ is introduced, based on a biaromatic atropisomeric core, which is advantageously obtainable in enantiopure form without HPLC separation steps by a synthetic route hinging on enantiopure 2,2’-dibromo-1,1’-binaphthalenes. The antipodes of the new inherently chiral monomer can be easily electrooligomerized, yielding inherently chiral electrode surfaces that perform well in both cyclic voltammetry (CV) enantiodiscrimination tests with pharmaceutically interesting molecules and in magnetoelectrochemistry experiments.

## 1. Introduction

**Figure 1:**
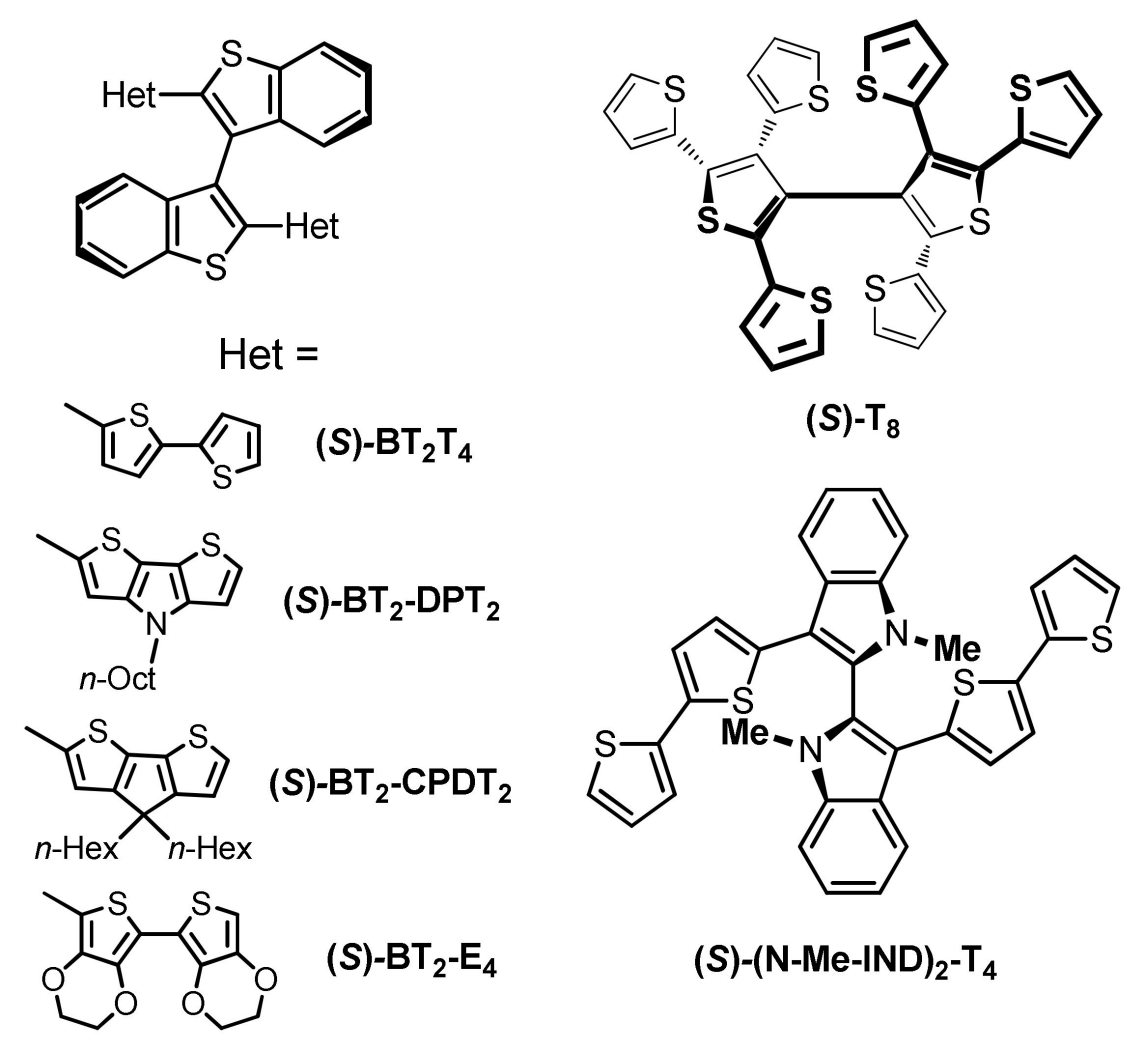
The three families of inherently chiral monomers.

Enantioselective electroanalysis is a very attractive target, potentially enabling discrimination between enantiomers of electroactive chiral probes without preliminary separation steps. While many proposed approaches appear irresolute so far [1,2], inherently chiral electroactive monomers based on atropisomeric biheteroaromatic cores and oligothiophene wings have recently been shown to be excellent starting materials for the electrodeposition of electrode surfaces consisting of inherently chiral oligomers. These materials have outstanding enantiodiscrimination ability for chiral electroactive probes in terms of large potential differences in voltammetry experiments [2]. In particular, three families have been explored so far (Figure 1), based on:

- a 3,3′-bibenzothiophene core, including **BT_2_T_4_** [3,4], **BT_2_CPDT_2_** and **BT_2_DTP_2_** [5], **BT_2_E_4_** [6];
- a 3,3′-bithiophene core, including all-thiophene spider-like **T8_3_** [7];
- a 2,2′-biindole core, including **(N-Me-IND)_2_-T_4_** [8].

In spite of their outstanding properties, a common drawback is that since all of the above monomers have been synthesized as racemates, expensive semi-preparative HPLC resolution on a chiral stationary phase was necessary to obtain them in the enantiopure form required to prepare the chiral electrode surfaces.

**Figure 2:**
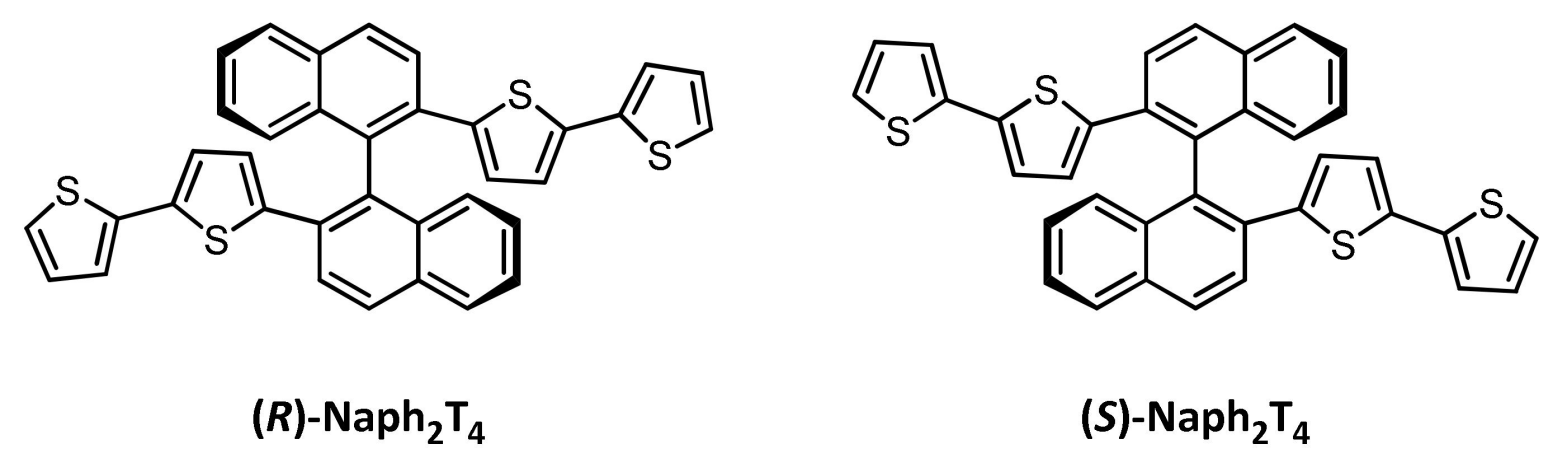
The two antipodes of BT_2_T_4._

In this context, we propose the new inherently chiral monomer **Naph_2_T_4_** (Figure 2), based on a biaromatic carbocyclic rather than biheteroaromatic atropisomeric core. This monomer can be can be obtained in enantiopure form by a synthetic route hinging on 1,1′-binaphthyl-2,2′-diamine as a chiral intermediate, commercially available in both enantiopure forms at reasonable price, or synthesizable as a racemate [9] and easily resolvable by fractional crystallization of the diastereomeric salts with enantiopure chiral acids [10]. 

**Figure 3:**
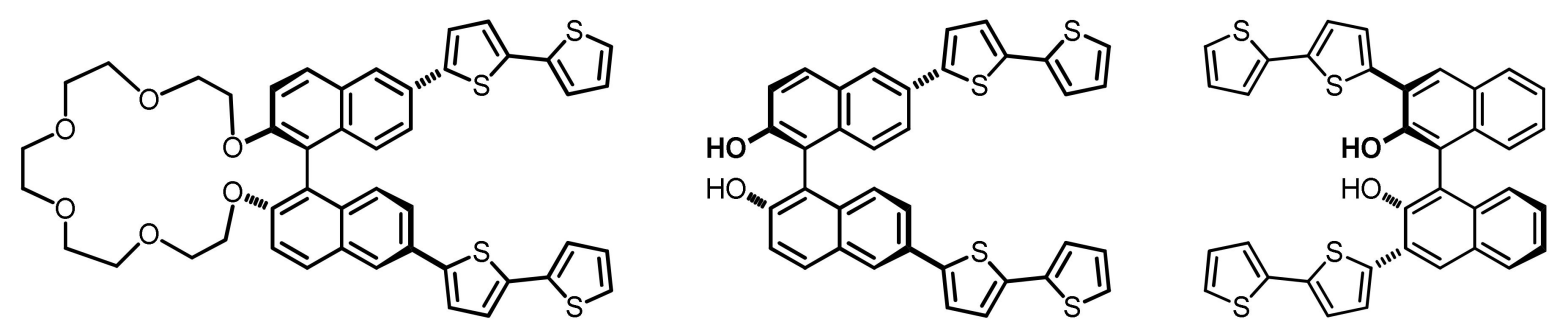
Examples of chiral electroactive monomers based on binol.

Several binaphthyl monomers had already been considered a few years ago, yielding promising performances. After an interesting preliminary study comparing electrooligomerization, spectroelectrochemistry and in situ conductance in two binol derivatives [11], Song and coworkers tested films obtained from a 1,1’-binaphthyl-2,2’-crown-ether derivative with 2,2’-bithiophene terminals in the 6,6’-position, and from a 2,2’-dihydroxy-1,1’-binaphthalene derivative with the same terminals in the 3,3’- or in 6,6’- positions (Figure 3), [12]. Very interesting results were obtained with the binol derivatives tested against chiral 2-amino-2-phenylethanol, which specifically interacted with the binol hydroxy groups as revealed by film conductivity decrease or by the film oxidation CV peak current decreasing with the concurrent appearance of a new peak at less positive potentials [12]. Even more attractively, significantly different potential trends were observed when testing the films in potentiometric mode with the two enantiomers of 1-(1-naphthyl)-ethylamine at increasing concentrations, which was explained by the authors in terms of enantiodiscrimination by the dissymmetric atropisomeric scaffold [12]. The difference in potential vs. concentration trends observed by Matsuda and Miyashita for the phenylethylamine enantiomers on electrodes modified with Langmuir–Blodgett films featuring binol pendants were also remarkable [13].

It is worth observing that unlike Song’s monomers, the connectivity of our present **Naph_2_T_4_** monomer enables efficient conjugation over the whole backbone, although this is attenuated by the torsional angle, which makes the monomer truly inherently chiral, i.e., the coincidence of the electroactive backbone with the stereogenic element.

In this work, chiral electrodes prepared by enantiopure **Naph_2_T_4_** electrodeposition are characterized and studied in chiral voltammetry enantiodiscrimination tests (considering tryptophan besides our usual probes *N*,*N’*-dimethyl-1-ferrocenylethylamine and 3,4-dihydroxyphenylalanine (DOPA) as well as magnetoelectrochemistry experiments. Their performances are discussed in comparison to those of the above selectors based on biheteroaromatic atropisomeric cores.

## 2. Results and Discussion

### 2.1. Monomer Synthesis

**Scheme 1:**
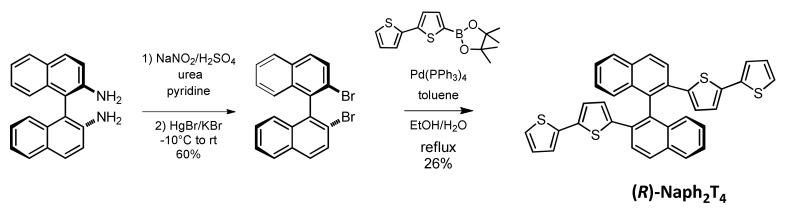
Relevant steps for the synthesis of (*R*)-Naph_2_T_4._

The key step in the synthesis of **Naph_2_T_4_** (Scheme 1, referring to the (*R*)-antipode case) is the functionalization of the atropisomeric core that was performed through a Suzuki Pd-mediated coupling reaction starting from the enantiopure 2,2’-dibromo-1,1’-binaphthalene, which was identified as the intermediate of choice due to its accessibility [14].

The (*R*)- and the (*S*)-1,1’-binaphthalene-2,2’-diamine were converted into the double diazonium salts that were then transformed into the corresponding dibromoderivatives according to a Sandmeyer-type reaction effected with KBr and HgBr_2_. The optical purity of the enantiomers was checked by HPLC on a chiral stationary phase and were found to be 98.5% and 96% for the (*S*)- and (*R*)-antipode, respectively.

The Pd-tetrakis-mediated Suzuki reaction, performed in a 3:1:1 mixture of toluene, water and ethanol in the presence of sodium carbonate as a base and with 5-(4,4,5,5-tetramethyl-1,3,2-dioxaborolan-2-yl)-2,2’-bithiophene afforded the enantiomers of Naph_2_T_4_, maintaining the enantiomeric purity of the corresponding dibromoderivatives and thus allowing us to obtain an inherently chiral monomer in an enantioselective way for the first time.

**Scheme 2:**
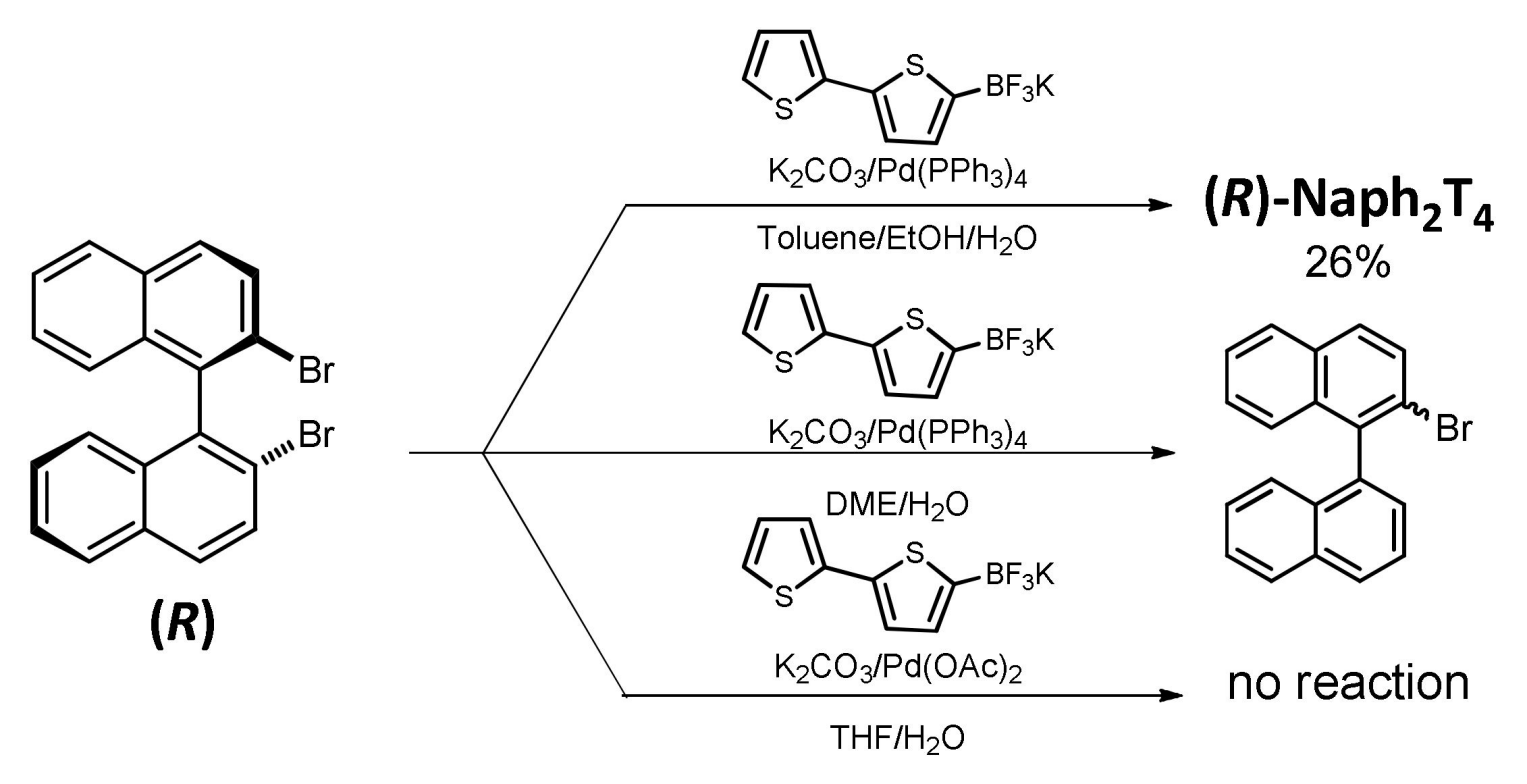
Synthesis of **Naph_2_T_4_** based on potassium bithiophenyltrifluoroboronate.

Unfortunately, the yields of the reaction were modest (25–27%) with 3-(2,2’-bithiophen-5-yl)-1,1’-binaphthalene being an expected, but unfortunately relevant, by-product (Scheme 2) [15].

In order to attempt to improve the outcome of the Suzuki reaction we used a more reactive boronic acid derivative, namely potassium bithiophenyltrifluoroboronate, [16] under identical experimental conditions, however this did not improve the final results. The outcome of the reaction completely changed as a result of using a 1:1 dimethoxyethane/water mixture as the solvent, since the 2-bromo-1,1’-binaphthalene was identified as the main reaction product. Also, the use of Pd(OAc)_2_ as a palladium source was found to be ineffective and the unreacted starting material was recovered after 12 h reaction time. It is evident that the above synthetic procedures are inadequate for applicative purposes and that a deeper screening of more efficient catalysts would be required [17,18,19]. However, we considered testing the performances of **Naph_2_T_4_** as a primary concern rather than the synthetic efficiency.

### 2.2. Computational Structural Study

#### 2.2.1. Racemization Barriers

**Figure 4:**
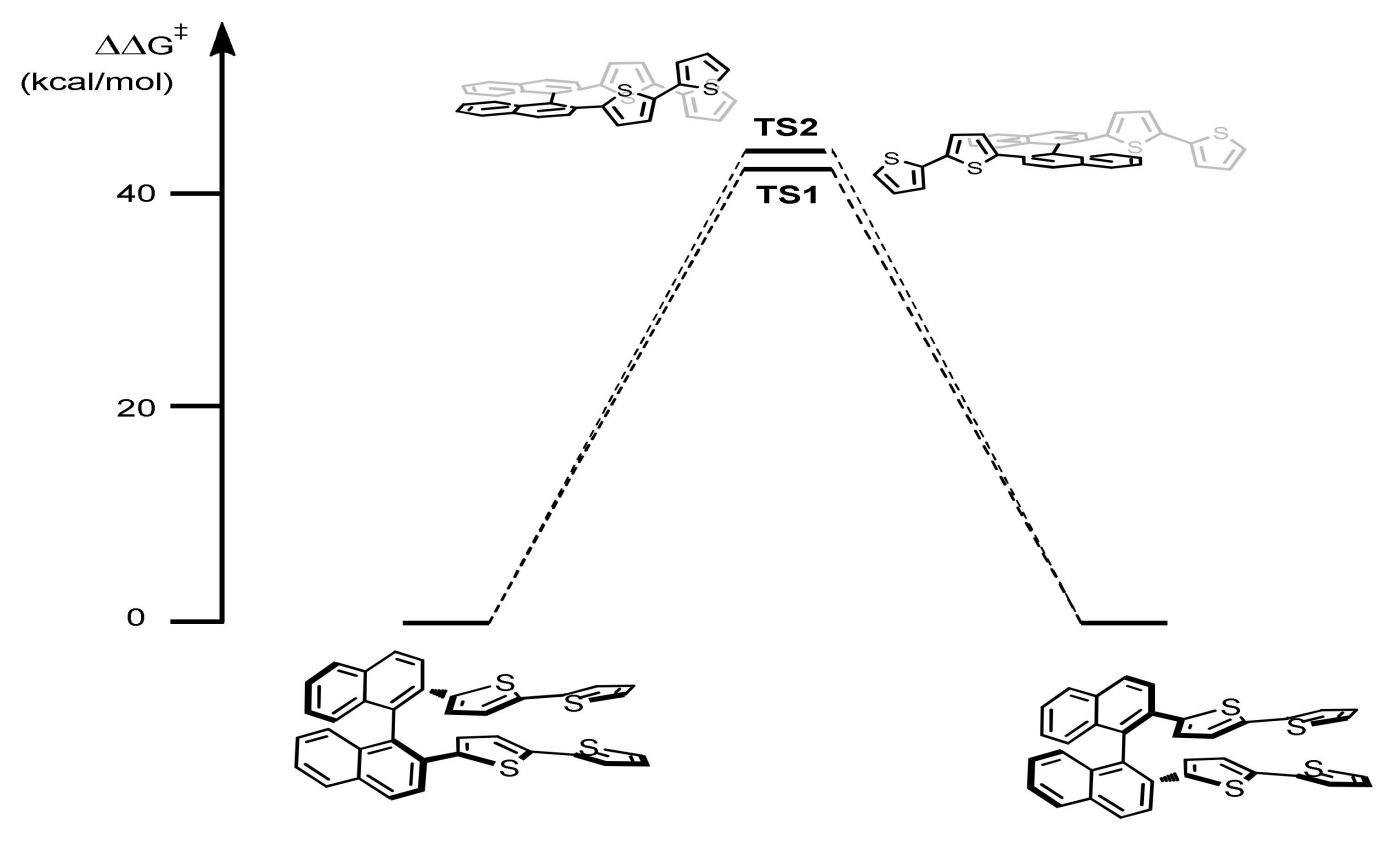
Reaction pathway for the racemization of **Naph_2_T_4_**. TS: transition state. ΔΔG^#^ is the difference between the free Gibbs energy of the TS and the free Gibbs energy of the fundamental state.

Since two different pathways and transition states for the configurational inversion of binaphthyl systems are known [20,21,22,23,24], the racemization process of **(*S*)-Naph_2_T_4_** was studied using a Dendity Functional Theory (DFT) approach. Computational studies (detailed in Section 3.2, as well as in Supplementary Materials Figure S1) point to the most stable monomer structure being the one with the bithiophene wings, in the preferred *s*-trans conformation, oriented in an orthogonal arrangement respective to the naphthyl rings (Figure 4). The torsional barrier around the bond interconnecting the naphthalene rings, calculated by B3LYP/6 − 311G + (3df,3pd)//B3LYP/6 − 31G(d,p) level from the ground energy level, is about 44 Kcal mol^−1^, confirming that the monomer has high configurational stability. Comparing an identical computational approach (Table 1) towards **(*S*)-Naph_2_T_4_** to the formerly developed biheteroaromatic scaffolds **(*S*)-(*N*-Me-IND)_2_T_4_** and **(*S*)-BT_2_T_4_** [3,8] shows that **Naph_2_T_4_** appears to be the one with the highest racemization barrier.

Two possible transition states (TS) for racemization were located for all three of the above compounds, i.e., TS2, related to a rotation process in which the two bithiophenyl substituents face together in a cisoid arrangement (*s*-*cis*) of the binaphthalene scaffold and TS1, where the two coplanar bithiophenyl chains face the naphthalene hydrogen atom in *peri* position in a transoid arrangement (*s*-*trans*). As shown in Table 1, TS1 is generally more accessible than TS2.

Theoretical calculations (Table 1) allowed us to determine some interesting geometrical parameters of the three monomers that were useful to rationalize their specific behavior in the oxidative oligomerization process and to attempt to give some explanation to their different enantiodiscrimination ability towards the same analyte (see below).

We considered a particularly meaningful structural parameter, the torsional angle, between the two halves of the atropisomeric biaromatic system (calculated at the fundamental state). We surprisingly discovered that these parameters are very different for the three monomers, ranging from 77.88° for **Naph_2_T_4_** to 117.71° for **(N-Me-IND)_2_T_4_**, while the **BT_2_T_4_** value (100.93°) lies exactly in the middle.

#### 2.2.2. Molecular Orbital Energies and Topologies from DFT Calculations

**Figure 5:**
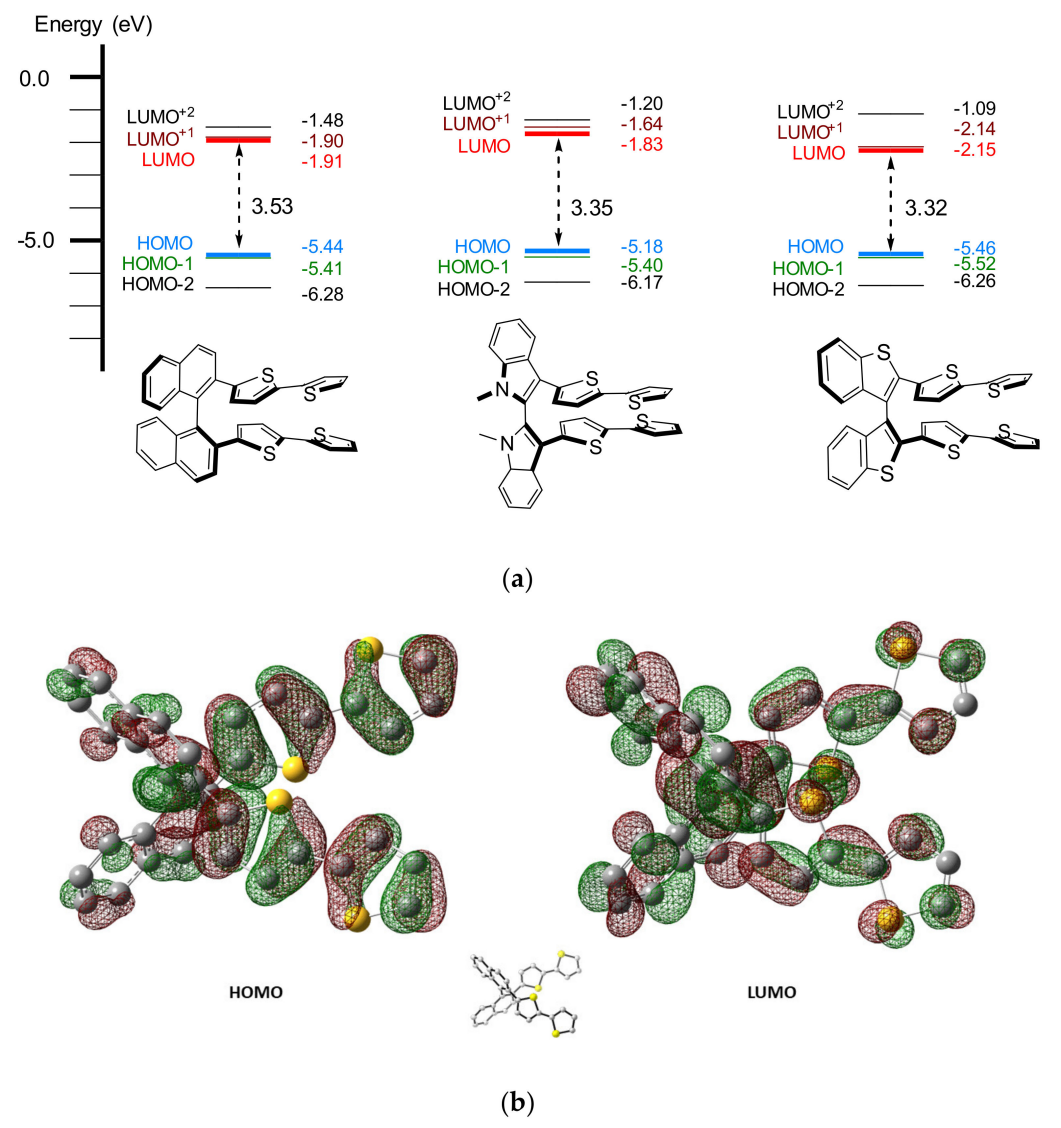
(**a**): DFT-calculated molecular orbital energies of **(*S*)-Naph_2_T_4_**, **(*S*)-(*N*-Me-IND)_2_T_4_** and **(*S*)-BT_2_T_4_**. (**b**): Frontier molecular orbital topologies for **(*S*)-Naph_2_T_4_**. Contours of **(*S*)-Naph_2_T_4_** molecular orbitals were plotted using Gaussview.

DFT calculations performed at th B3LYP/6 − 311G + (3df,3pd) // B3LYP/6 − 31G(d,p) level of theory under vacuum conditions yielded the molecular orbital energy levels summarized in Figure 5a for the three inherently chiral monomers.

**(N-Me-IND)_2_T_4_** features a Highest Occupied Molecular Orbital (HOMO) level much higher than the other two cases, consistent with the very electron rich biindole core on which the first oxidation must be mainly localized [8]. Instead, in **Naph_2_T_4_**, the HOMO orbital is mainly localized on the bithiophene wings (Figure 5b), and the same should apply for BT_2_T_4_; this is confirmed by the electrooligomerization taking place by cycling the potential around the first oxidation peak for the last two molecules (next paragraph and [3]) while in the bi-indole case, the bithiophene wings are not the first activated redox site [8].

Concerning Lowest Unoccupied Molecular Orbital (LUMO) levels, the **(N-Me-IND)_2_T_4_** level is higher than the BT_2_T_4_ one, consistent with the pyrrole-bithiophene system being richer in electrons than the terthiophene one; on the other hand, the **Naph_2_T_4_** level, mainly localized on the phenyl core (Figure 5b), is higher than the BT_2_T_4_ one, consistent with the phenyl-bithiophene system having a less effective conjugation with respect to terthiophene.

On the whole, **(*S*)-BT_2_T_4_** and **(*N*-Me-IND)_2_T_4_** feature similar energy gaps (although the second one is shifted to higher energies), which are significantly smaller than the **(*S*)-Naph_2_T_4_** one.

### 2.3. Electroactivity of the Naph_2_T_4_ Monomer

**Figure 6:**
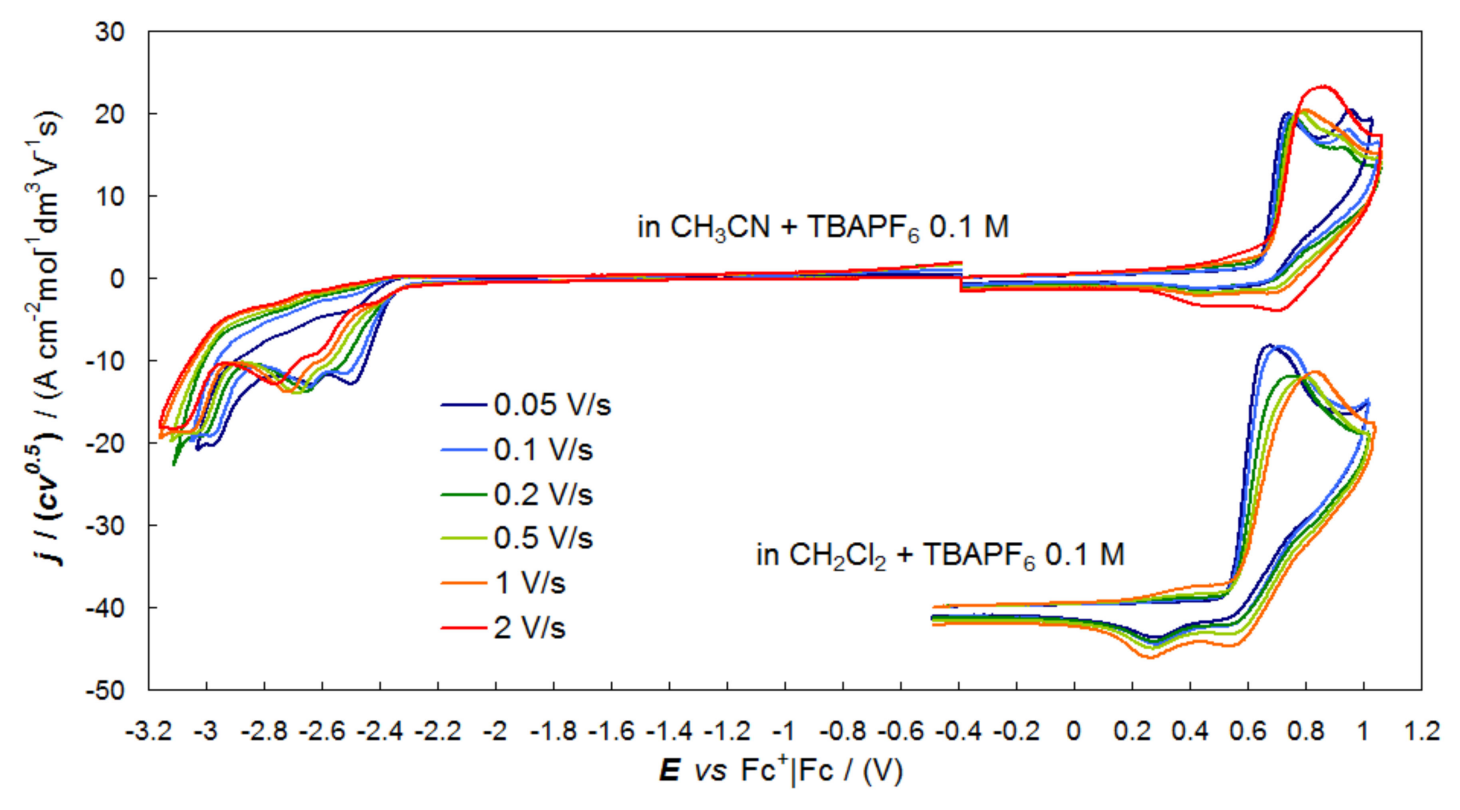
Normalized CV patterns of the **Naph_2_T_4_** monomer on Glassy Carbon (GC) electrode at scan rates ranging from 0.05–2 V in acetonitrile (CH_3_CN, above) or dichlorometane (CH_2_Cl_2_, below), in both cases with 0.1 M TBAPF_6_ as the supporting electrolyte.

The electroactivity of the **Naph_2_T_4_** monomer was studied by cyclic voltammetry at scan rates ranging from 0.05–2 V/s, in both acetonitrile and dichloromethane solvents (Figure 6).

In many former cases of inherently chiral monomers, a two-peak first oxidation system was often observed, accounting for the two equal molecule moieties behaving as equivalent and partially interacting redox centers. The twin peak potential difference increased with increasing reciprocal interaction (also dependent on solvent polarity); the more polar acetonitrile resulted in a screening effect with the two peaks becoming closer or even merging [8].

The computations reported in the former paragraph account for **Naph_2_T_4_** having the highest torsional barrier in the inherently chiral monomer series considered so far, which should result in very poor interactions between the two moieties. This is consistent with the observation of a single first oxidation CV peak (although larger than a canonical one and tending to split) rather than a well-defined two-peak system. It is worthwhile noting that in acetonitrile solution, the second oxidation peak observed at low scan rates does not correspond to a molecule moiety first oxidation, but to a further oxidation process of an electroactive product formed by chemical follow up, as evident from its disappearance upon increasing the potential scan rate. Moreover, consistently with the above observations on the solvent effect, in the less-polar dichloromethane, the two-merged-peak nature of the oxidation peak is already evident at low scan rates, while in the more-polar acetonitrile, it only becomes evident at higher scan rates because of the chemical follow up taking place at a low scan rate (Figure 6).

The reduction pattern can be only observed in acetonitrile, since the dichloromethane potential window is significantly narrower on that side on account of the solvent bulk reduction at a rather early potential. It features a first twin-peak system, their relative ratio changing with scan rate, and irreversible both chemically (no return peak, pointing to chemical follow up involving the electron transfer product) and electrochemically (large potential shift with increasing scan rate, pointing to high energy barrier for the electron transfer). Such a first twin-peak system is followed by a further single reduction peak located just before the background.

Both the nearly splitting first oxidation peak and the twin first reduction peak system can be assumed to involve a couple of equivalent redox sites, namely the two **NaphT_2_** moieties. However, as confirmed by the HOMO and LUMO maps resulting from DFT calculations (Figure 5), first oxidations should be more localized on the electron-richer bithiophene T_2_ wings, yielding radical cations that can undergo coupling and resulting in electrooligomerization (as reported in the next paragraph). Instead, first reductions, which should involve radical anion formation, should be more localized on the binaphtyl core moieties. The increased proximity of the redox centers might justify a twin-peak system rather than a completely merged/nearly splitting one. The −2.5/−2.8 V reduction potential range looks consistent with the reduction potential of unsubstituted naphthalene, located in acetonitrile at about −2.6 V vs Saturated Calomel Electrode (SCE); that is, at about −2.9 V vs the formal potential of the intersolvental ferricenium|ferrocene (Fc^+^|Fc) reference redox couple, recorded in the same conditions, with various tetraalkylammonium tetrafluoborate supporting electrolytes [24]), considering that the positive shift can be justified in terms of extended conjugation.

**Figure 7:**
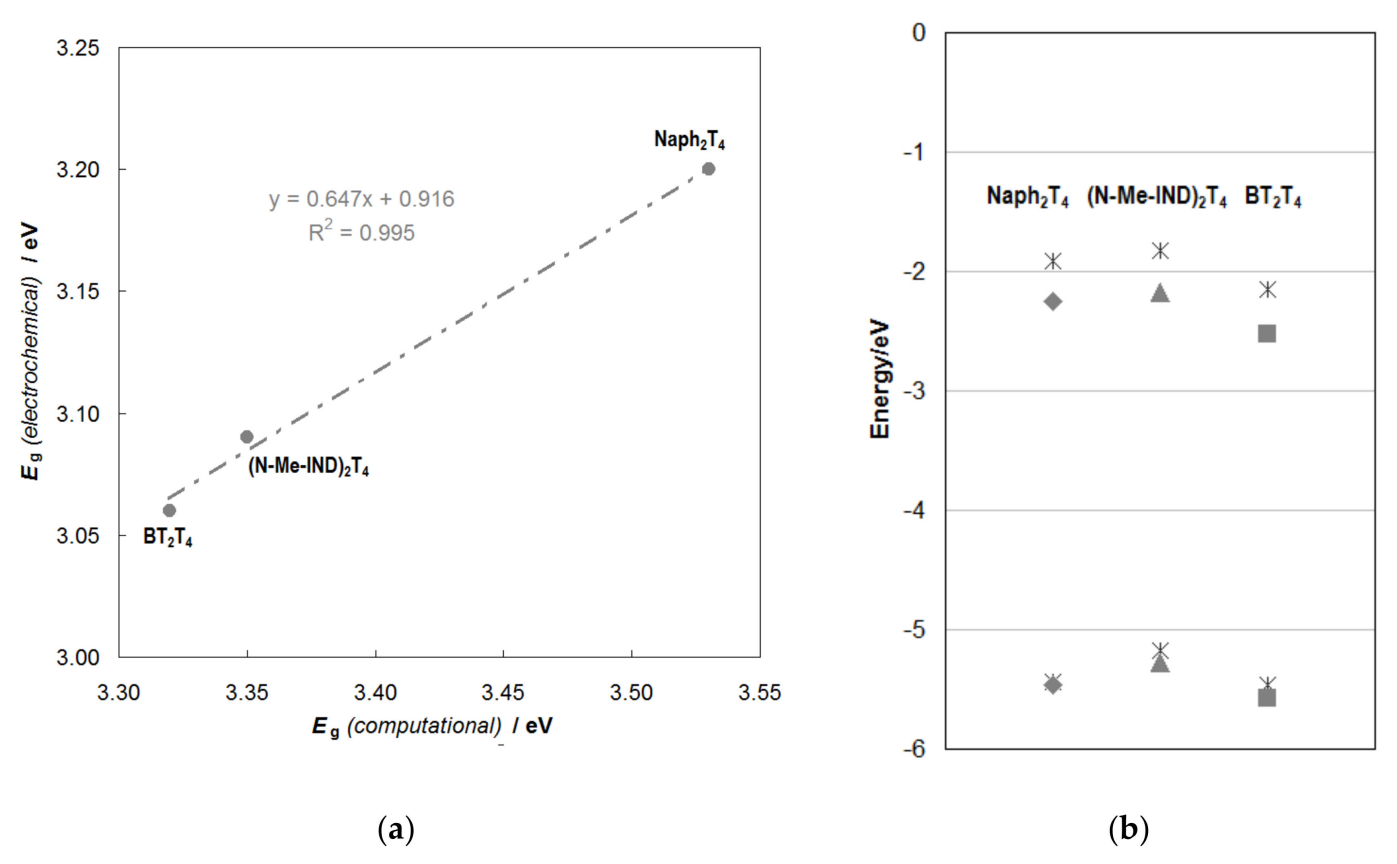
(**a**) Correlation between energy gaps (*E*_g_) calculated in a vacuum and observed in electrochemical experiments in acetonitrile with 0.1 M TBAPF_6_ as a supporting electrolyte, for **Naph_2_T_4_** (this work), **(N-Me-IND)_2_T_4_** [8] and **BT_2_T_4_** [21]. (**b**) Calculated (full symbols) and electrochemical (asterisk) HOMO and LUMO levels in the three cases.

Notably, a very good correlation exists (Figure 7a) between energy gaps (*E*_g_) calculated in a vacuum and observed in electrochemical experiments in acetonitrile with 0.1 M TBAPF_6_ as the supporting electrolyte for **Naph_2_T_4_**, **(N-Me-IND)_2_T_4_** [8] and **BT_2_T_4_** [25]. A good correlation also exists between single level *E*_HOMO_ and *E*_LUMO_ trends (Figure 7b), with the electrochemical energy levels being estimated according to *E*_HOMO_ = −e × (*E*_pIa_ vs Fc^+^|Fc + 4.8 V) and *E*_LUMO_ = −e × (*E*_pIc_ vs Fc^+^|Fc + 4.8 V) [26]. It is true that ***E***_HOMO_ are nearly coincident, while *E*_LUMO_ are systematically lower in the experimental case; however, it must be underlined that electrochemical data are obtained in a solvent (here a polar one), enabling high stabilization of charged species, and in the presence of a supporting electrolyte, potentially resulting in ionic couple formation, besides being calculated according to an estimated value of the Fc^+^|Fc couple redox potential in a vacuum. For this reason, trend consistency in systematic series should be looked for, rather than coincidences of single energy levels.

### 2.4. Electrodeposited Oligo-Naph_2_T_4_ Inherently Chiral Electroactive Films

Enantiopure oligomer films can be easily obtained from the corresponding monomers, which undergo very fast and regular electrooligomerization both in CH_3_CN and CH_2_Cl_2_ with TBAPF_6_ 0.1M as the supporting electrolyte. In particular, films were prepared by cycling 36 times around the first oxidation peak, corresponding to the formation and coupling of radical cations mainly localized on the **Naph_2_T_4_** thiophene terminals, which have free α positions, according to the typical mechanism described by Heinze et al. [27] Both films grown in CH_3_CN and CH_2_Cl_2_ show good stability upon subsequent potential cycling in monomer-free solution, as required for use as enantioselective electrode surfaces.

**Figure 8:**
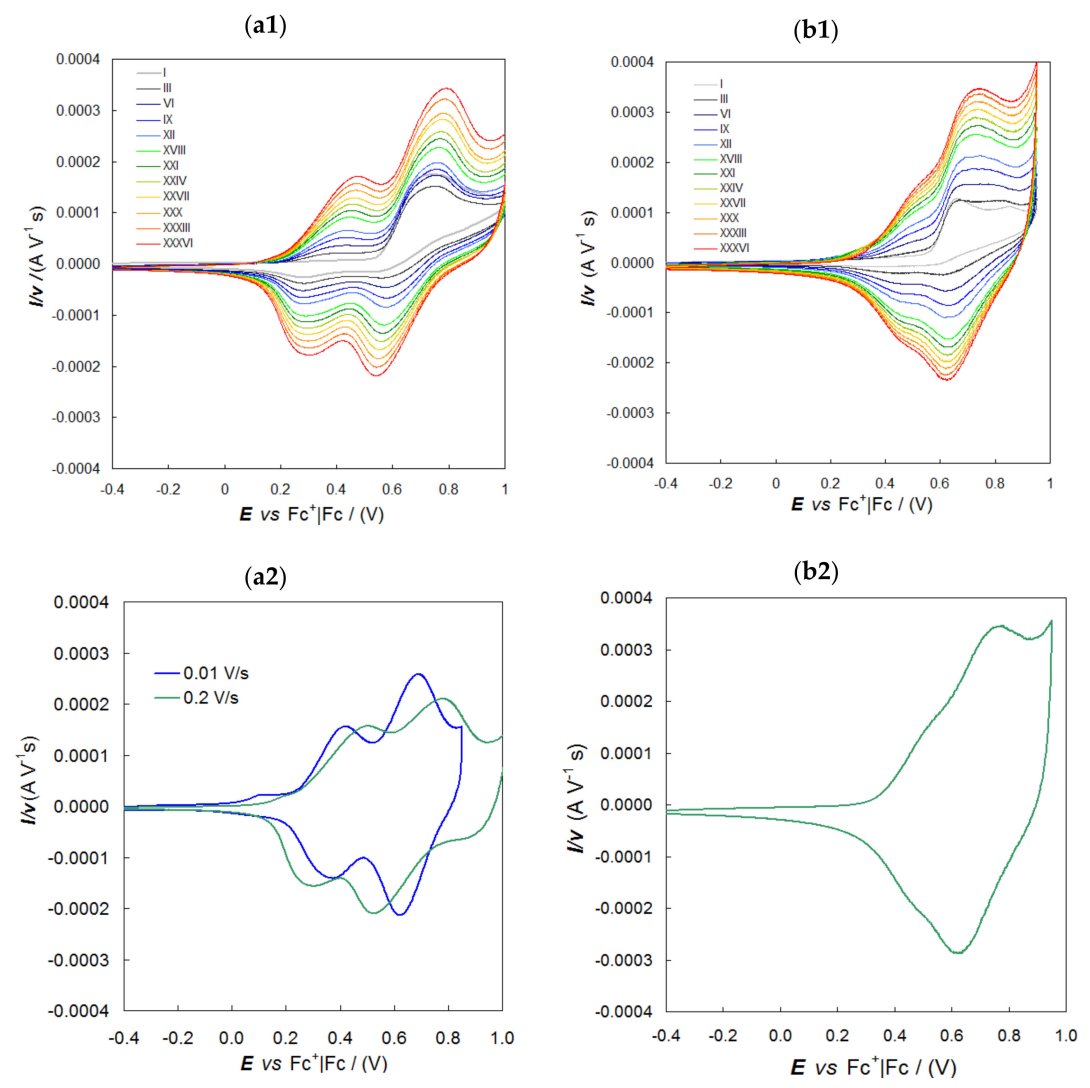
Electrooligomerization of 0.00075 M **(*R*)-Naph_2_T_4_** monomer on a GC tip electrode in dichlorometane (CH_2_Cl_2_) (**a1**) and acetonitrile (CH_3_CN) (**b1**). In both cases, 0.1 M TBAPF_6_ was used as the supporting electrolyte and oxidative potential cycling was carried out at 0.2 V s^−1^. The last stability cycle in monomer-free solution, in CH_2_Cl_2_ (**a2**, comparing two different scan rates) and in CH_3_CN (**b2**, at 0.2 V s^−1^ potential scan rate).

Oligomerization and stability CV patterns (Figure 8) show that growth rate is comparable in the two media (note that the figures have the same scale), but in the CH_2_Cl_2_ case, the film oxidation is easier, since its onset is located at significantly less positive potentials. 

The potential scales have been duly normalized against the intersolvental reference couple ferrocene (this eliminates the solvent effect on the reference electrode liquid junction; thus, the residual potential differences can be attributed to the solvent effect on the process). Moreover, in the CH_2_Cl_2_ case, two well-defined redox sites are observed for the electroactive films (nicely reversible, especially at low rates), while they are nearly merged in the acetonitrile case. However, the global charge associated with the stability cycle (provided by integration of the CV peak systems) is comparable in the two cases.

Remarkably, and unlike former biheteroaromatic cases, the oligomer films have different colors according to the growth medium, i.e., yellow for CH_2_Cl_2_ and green for CH_3_CN.

Unfortunately, investigation of the oligomer nature by MALDI analysis was prevented by very low film solubility, unlike the parent case of **oligo-BT_2_T_4_** films [4]. However, analogously to the **BT_2_T_4_** case, **Naph_2_T_4_** films might include both cyclic and open oligomers; indeed, oligonaphtalenes have been recently reported in the literature [28]. Moreover, a FeCl_3_ oligomerization reaction was carried out and the Soxhlet tetrahydrofuran extracts were analyzed by Laser Desorption Ionisation (LDI), yielding evidence of the presence of cyclic dimers (mainly) and trimers (Supplementary Materials Figure S2).

The surface morphology of the two films was characterized by tapping mode atomic force microscopy (AFM). AFM investigation allowed us to obtain both information on the film morphology at a nanometer level and to quantitatively evaluate their surface roughness.

**Figure 9:**
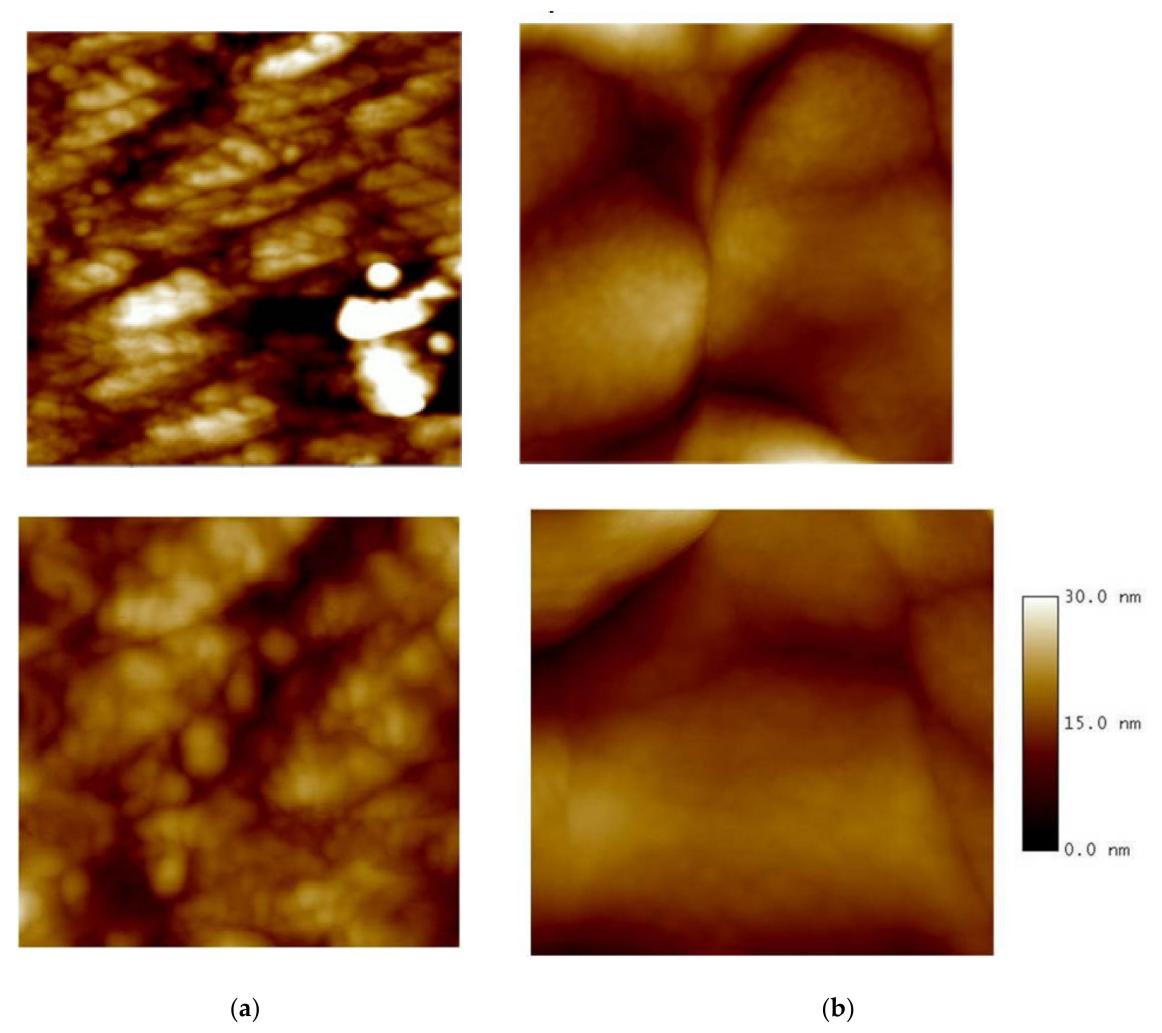
A comparison between AFM topography images of **oligo-(*R*)-Naph_2_T_4_** films grown in CH_2_Cl_2_ (**a**) and in CH_3_CN (**b**). Scan areas: both images on first row: 1 × 1 μm^2^; both images on second row: 500 × 500 nm^2^. Vertical (color) scale: 30 nm.

As an example, a comparison of AFM topography images of the **oligo-(*R*)-Naph_2_T_4_** films electrodeposited in CH_2_Cl_2_ and in CH_3_CN is reported in Figure 9.

In the AFM images of the **oligo-(*R*)-Naph_2_T_4_** sample grown in CH_2_Cl_2_, a uniform globular structure is observed, with particle sizes of the order of tens of nanometers. The surface morphology significantly changes for the **oligo-(*R*)-Naph_2_T_4_** sample grown in CH_3_CN. In fact, as revealed by AFM imaging, the surface appears to be constituted of larger structures with, on average, dimensions of hundreds of nanometers.

At the nanometer level, both the studied films show a flat surface. Their root mean square (rms) surface roughness, obtained by analyzing several AFM topography images collected in different regions on the sample, was calculated according the following equation:

(1) $${\mathrm{rms}}_{\mathrm{xy}}=\sqrt{\sum_{x,y=1}^{N}\frac{{\left(Zx,y-Z\mathrm{average}\right)}^{2}}{N^{2}}}$$

where Z_average_ is the average Z value within the examined area, Z_x,y_ is the local Z value, and N indicates the number of points within the area.

The rms surface roughness values, measured on 500 × 500 nm^2^ areas, for the two studied films are reported in Table 2.

### 2.5. Enantiodiscrimination Voltammetry Tests 

Due to the high configurational stability of inherently chiral **Naph_2_T_4_** antipodes, films electrodepo sited from enantiopure monomers entirely retain their configuration, resulting in the corresponding enantiopure inherently chiral **(*R*)-** or **(*S*)-oligo Naph_2_T_4_** films.

The enantiodiscrimination ability of enantiopure films grown in dichloromethane was studied by testing them as electrode surfaces for the electroanalytical discrimination of enantiopure probes without preliminary separation steps. In particular, CV tests were carried out at 0.05 V s^−1^ on GC electrodes, considering three chiral electroactive molecular probes of different chemical natures and electrochemical reactivities.

Our usual "standard" chiral probe, *N*,*N*′-dimethyl-1-ferrocenylethylamine (FcA), was tested in dichloromethane with 0.1 M TBAPF_6_ as the supporting electrolyte, resulting in an irreversible first oxidation CV peak at potentials only slightly different for the two enantiomers (unlike the benchmark **oligo-BT_2_T_4_** case), while larger potential differences are observed for the second oxidation peaks (Figure S4 in Supplementary Materials 3).

**Figure 10:**
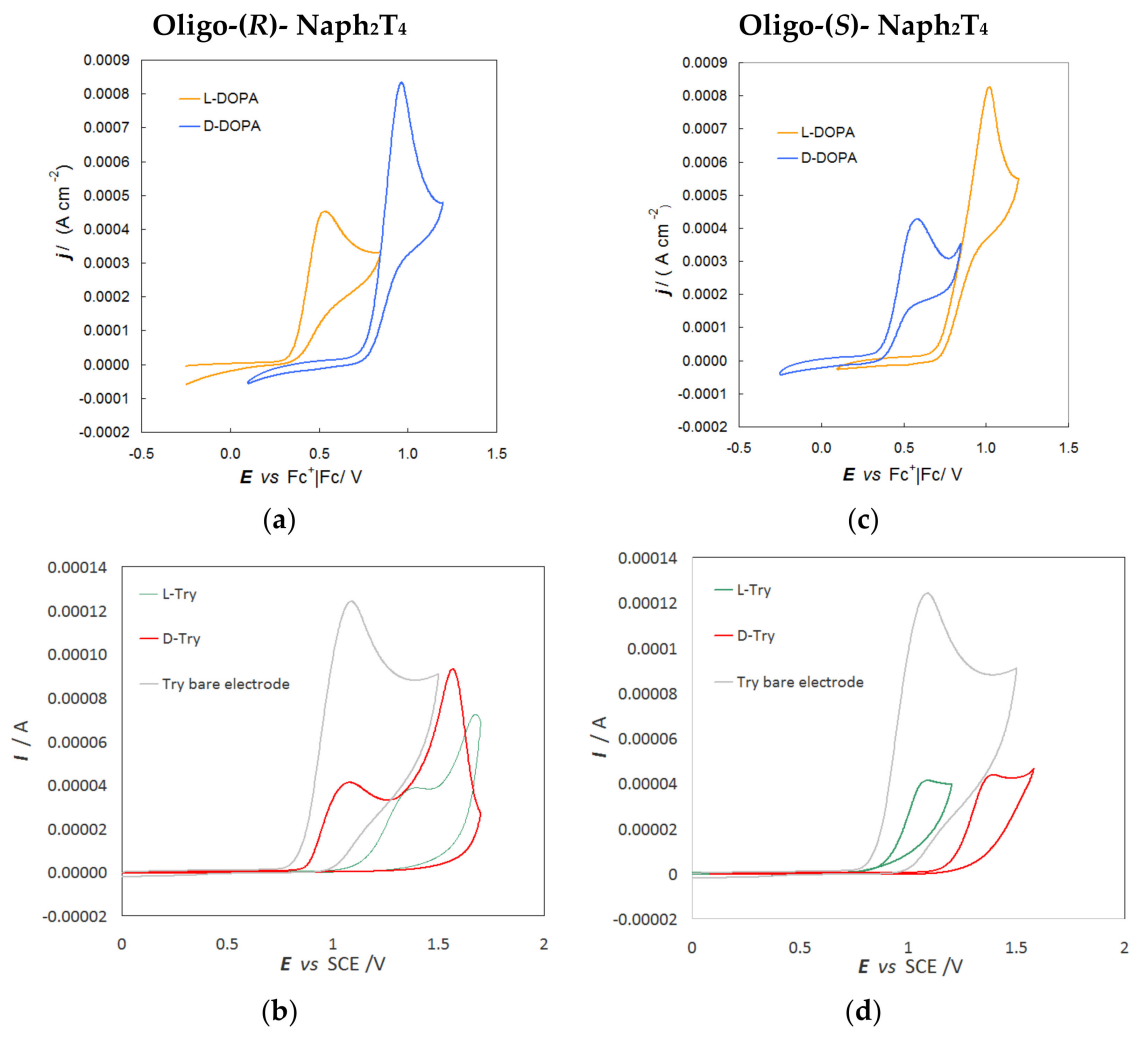
Enantiodiscrimination CV tests in aqueous solution with 0.05 M HCl as the supporting electrolyte, performed on a GC electrode modified with **oligo-(*R*)-Naph_2_T_4_** and **oligo-(*S*)-Naph_2_T_4_** enantiopure films (**a**,**b**,**c**,**d**) electrodeposited in dichloromethane, with the enantiomers of two chiral probes: (l)- and (d)-DOPA ((**a**,**c**), yellow and blue lines respectively) and (l)- and (d)-tryptophan (Try) ((**b**,**d**), green and red lines respectively; in the (*R*)-film case, the second oxidation peaks of the chiral probes are also reported).

The tests performed in aqueous solution with 0.05 M HCl as the supporting electrolyte with the enantiomers of two chiral probes of biological/pharmacological interest were quite remarkable, i.e., L- and D-DOPA (Figure 10a,c) and l- and d-tryptophan α-amino acid, an important new entry in our chiral probe gallery (Figure 10b,d).

In both cases, the CV peaks observed for the enantiomers (neatly specular in shape and potential upon inversion of probe or selector configuration) have huge potential differences, i.e., more than 0.4 V in the DOPA case (much more than in the benchmark **oligo-BT_2_T_4_** case), and about 0.4 V in the tryptophan case. Interestingly, in the DOPA case, significant differences are also observed in the antipode peak shapes, pointing to different mechanisms, which could be consistent with the large energy difference between the two processes. However, a large peak potential difference can be considered a sufficient, rather than necessary, condition for mechanism changes; in the tryptophan case, the two enantiomer peaks have the same shape notwithstanding a large potential difference. It is also interesting to observe that the peaks recorded on the filmed electrode are significantly smaller than those recorded on the bare one.

A very preliminary comparative test was also performed on the different enantioselection abilities exhibited by enantiopure oligo-**Naph_2_T_4_,** oligo-**BT_2_T_4_**, and oligo-**(N-Me-IND)_2_T_4_** films in terms of potential differences observed under the same operating conditions for the enantiomers of DOPA alongside those of the standard reversible probe *N*,*N’*-dimethyl-1-ferrocenylethylamine (Fca), namely (Table 3).

Considering that the electroactive films differ not only for the chemical structure of the monomer but also for the distribution of closed/open chain oligomers, and that the operating medium could also significantly modulate selector/probe interactions, it is difficult to clearly attribute the different enantiodiscrimination performances to a specific property. However, considering that the three monomers have quite a highly similar structure, except for the nature of the biheteroaromatic core, the dihedral angle can be discussed as a very important parameter for the film functional properties, including enantiorecognition.

In particular, it is intuitive that the two bithiophenic wings in monomers endowed with a narrow dihedral angle are in a much more favorable situation to form cyclic dimers than in monomers where they diverge. This observation is in agreement with the evidence supplied by MALDI experiments (assuming chemical and electrochemical oligomerization outcomes to be essentially consistent): the oligomers resulting from **Naph_2_T_4_** are nearly exclusively constituted by the cyclic dimer (Supplementary Materials 2), while **(N-Me-IND)_2_T_4_** affords both cyclic and open chain oligomers, with the latter largely prevailing. **BT_2_T_4_** oxidation produces a mixture, with the macrocycles prevailing on the open chain oligomers [4,29].

In this light, a justification could be found for **oligo-Naph_2_T_4_** giving outstanding results only with small sized analytes, like α-aminoacids, while the open wings characterizing **oligo-(N-Me-IND)_2_T_4_** could be similarly effective in hosting small (DOPA) and large analytes (FcA), producing very good enantioselectivity levels in all cases. At the same time, **oligo-BT_2_T_4_** looks like a very versatile material, generally exhibiting good, though not exciting, enantioselection performances, at least in the tested operating conditions.

### 2.6. Magnetoelectrochemistry Experiment

Chiral films obtained by electrooligomerization of **(*R*)-** or **(*S*)-Naph_2_T_4_** monomers could also provide attractive molecular spin selectors, consistent with the chiral-induced spin selectivity theory developed by Naaman and collaborators [30]. In this context, we tested enantiopure **oligo-Naph_2_T_4_** films in a magnetoelectrochemistry experiment, applying a protocol recently proposed by us [31], based on a modification of the spin dependent electrochemistry (SDE) protocol by Naaman’s group, and recently yielding striking effects on various chiral and inherently chiral electrodeposited electrode surfaces [8,30,31].

According to the protocol, an achiral reversible redox couple is studied by cyclic voltammetry on a non-ferromagnetic working electrode (ITO), either bare or covered by a very thin enantiopure chiral film (obtained with a single oligomerization cycle), with or without application of an external permanent magnet, which can be flipped to change the magnetic field orientation. 

**Figure 11:**
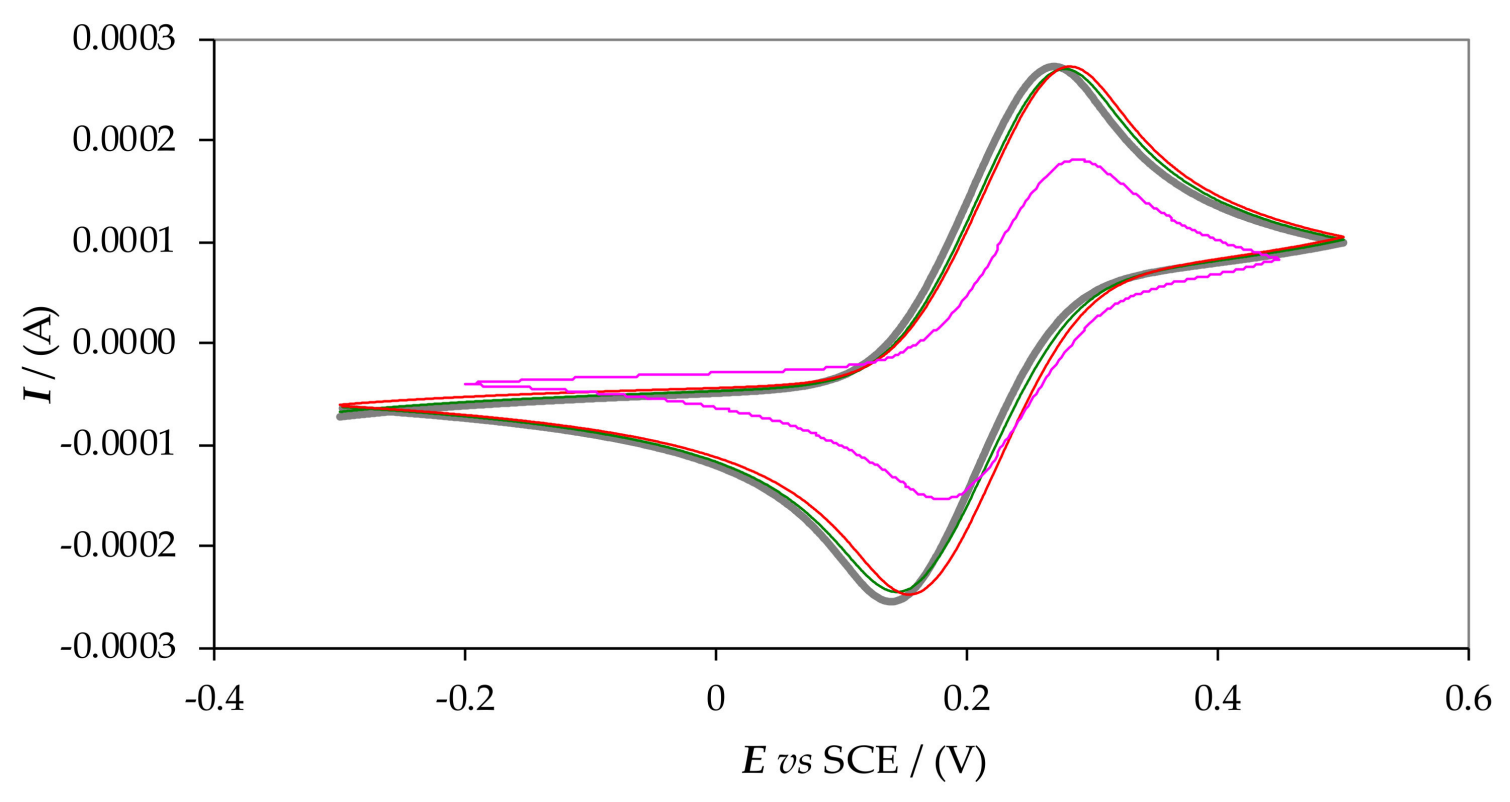
CV patterns recorded at 0.05 V s^–1^ for the achiral couple K_3_[Fe(CN)_6_] + K_4_[Fe(CN)_6_] (each of them 2.5 mM in 0.4 M KCl): on a bare Indium Tin Oxide (ITO) electrode without the application of an external magnet (grey line); on bare ITO as a function of the magnetic field orientation (green line for north pole, red line for south pole); on hybrid ITO|oligo-(*S*)-Naph_2_T_4_ electrode (pink line) without the external magnet.

As shown in Figure 11, the CV peaks of the achiral Fe(III)/Fe(II) redox probe on the bare ITO surface do not change as a function of the magnetic field orientation, neither in terms of potential nor of current. A current variation, consistent with the modified electrode surface, is observed on the ITO electrode modified with an enantiopure **oligo-(*S*)-Naph_2_T_4_** in the absence of an applied magnetic field, while the potential remains nearly unchanged with respect to the bare electrode, although in principle, both current and potential could change by electrode surface modification.

**Figure 12:**
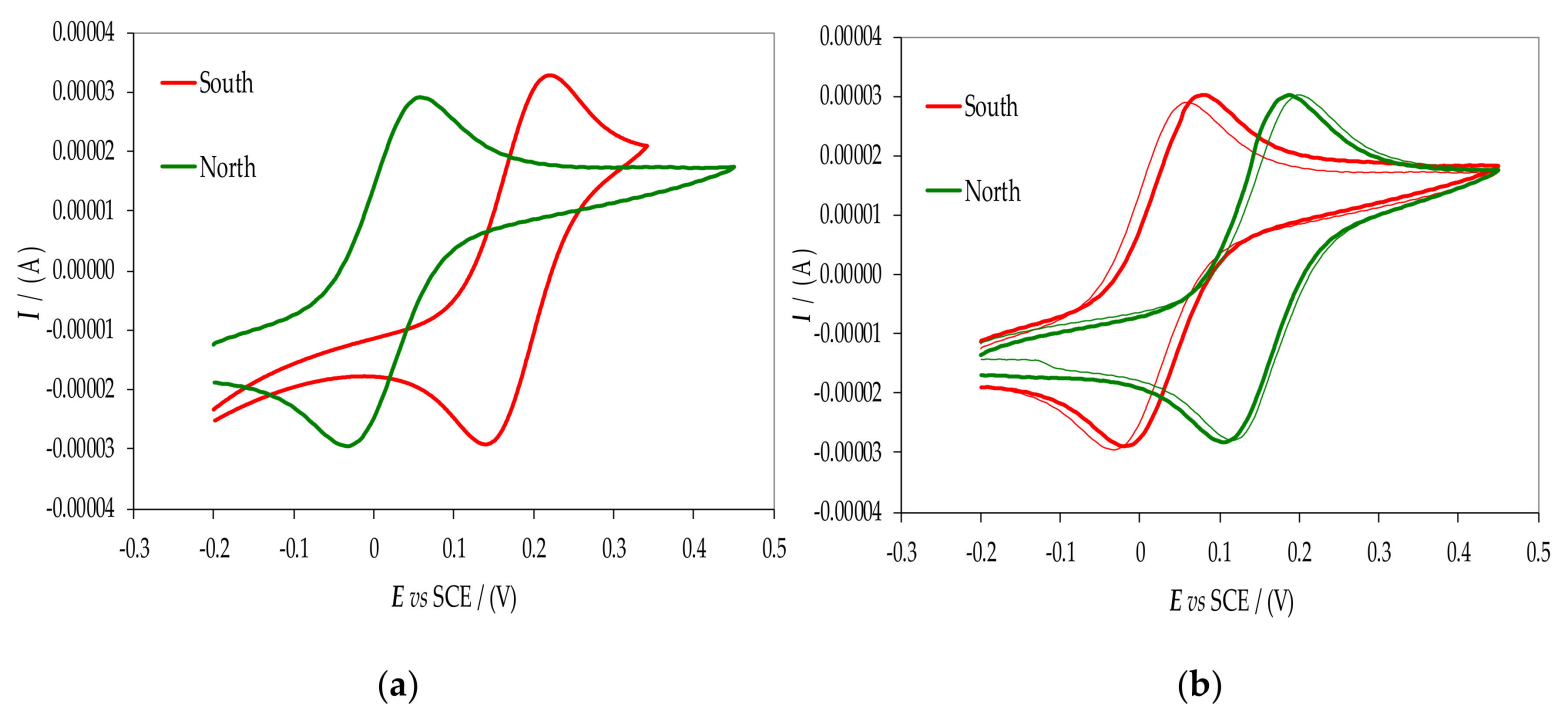
CV patterns recorded at 0.05 V s^–1^ for the Fe(III)/Fe(II) achiral couple on hybrid ITO|**oligo-(*R*)-Naph_2_T_4_** (**a**) and **oligo-(*S*)- Naph_2_T_4_** (**b**) electrodes as a function of the applied magnetic field orientation (red line vs green line for south and north configuration, respectively). In the plot on the right, reproducibility tests are also shown.

By combining both electrode modification with the enantiopure chiral thin film and application of the external magnet, the CV peaks of the achiral Fe(III)/Fe(II) redox probe show impressive peak potential shifts upon mechanically flipping the magnet orientation along its magnetic axis (north vs. south magnet pole), specularly upon changing the film (*R*)- or (*S*)-configuration (Figure 12). 

In other words, the achiral redox couple under a magnetic field behaves like a chiral redox couple, just as achiral chromophores can give circular dichroism, i.e., behaving like chiral chromophores under the application of an external magnetic field. Many parallelisms and connections exist between chiral voltammetry and chiroptical spectroscopy, as we recently showed.

The striking effect is comparable to that recently observed with other inherently chiral films [8,31,32]. Although interpretation has still to be assessed, it can be noticed that:

- magnetic fields result in splitting of the α and β electron spin energies proportional to the magnetic field force, implying unbalance of α and β electrons;
- a mere rigid translation of chemically and electrochemically reversible CV signals implies an energy difference;
- an electrode process on a bare electrode under a NS or SN magnetic field represents a couple of specular and therefore energetically equivalent situations;
- the same applies to the process in the absence of the external magnetic field but on the electrode modified with (*R*)- or (*S*)- chiral films (which, according to Naaman’s theory, correspond to specular "internal" chiral magnetic fields);
- instead, a combination of the "internal chiral" with the "external" magnetic field results in two couples of specular situations, reciprocally in diastereomeric (i.e., energetically different) conditions.

An electron in rototranslational movement perpendicularly to an electrode interphase is a truly chiral object, and as such, its α and β enantiomers should be discriminated by the "internal" chiral magnetic field of a chiral film on the electrode surface; however, the two alternatives should be energetically equivalent unless a second magnetic field (the external one) is applied, either in a synergic or antagonist way. A problem is that the energy differences usually associated to spin effects are much smaller with respect to those implied by the observed potential difference. We look forward to deepening our investigation to achieve further clues.

## 3. Materials and Methods

### 3.1. Synthesis

#### 3.1.1. Synthesis of (R)-(+)-2,2’-dibromo-1,1’-binaphthalene

Concentrated H_2_SO_4_ (68 mL) was cooled to −10 °C and NaNO_2_ (5 eq., 2.1 g, 30 mmol) was slowly added under stirring. After the addition, the solution was allowed to come to room temperature until the NaNO_2_ was dissolved. Then, it was cooled to between −5 and −10 °C. A solution of (*R*)-1,1’-binaphthyl-2,2’-diamine (1 eq., 1.7 g, 5.98 mmol) in pyridine (14.6 mL) prepared previously was added dropwise to the solution below −5 °C. The resulting dark solution was stirred for an additional hour. Small chunks of ice were slowly added to the mixture under stirring to keep the temperature below −5 °C. The addition of ice was continued until no increase in temperature was observed. An ice-cooled mixture of urea (5 eq., 1.8 g, 30 mmol) in water (44 mL) was then added dropwise, maintaining temperature below −5 °C; persistent foaming was observed during the addition. When addition was complete, the solution was stirred for further 30 minutes.

A cold solution of mercury bromide (2.8 eq., 6.03 g, 16.74 mmol) and KBr (8.5 eq., 6.05 g, 51 mmol) in water (40 mL) was then added to the reaction mixture, causing the immediate formation of a light brown precipitate that was collected by filtration. The brown residue was grinded with KBr (10 eq, 7.14 g, 60 mmol) until a fine, intimate mixture was obtained. This mixture was dried in a round bottomed flask under vacuum and heated at 95 °C (0.1 torr) for 2 h. The resulting residue was extracted with CH_2_Cl_2_. The organic phase was then washed with aqueous sodium bisulfide, dried over MgSO_4_, filtered, and the solvent removed under reduced pressure. The residue was purified by column chromatography (*n*-hexane:CH_2_Cl_2_ = 9:1) to afford the desired product as a yellow solid.

Yield: 1.49 g (60%); ^1^H-NMR (CDCl_3_, 300 MHz): δ = 7.95 (d, *J* = 9.0 Hz, 2H), 7.89 (d, *J* = 9.6 Hz, 2H), 7.53 (d, *J* = 10.0 Hz, 2H), 7.52 (dd, *J* = 7.7 Hz, *J* = 1.3 Hz, 2H), 7.33 (dd, *J* = 7.7 Hz, *J* = 1.6 Hz, 2H), 7.11 (d, *J* = 9 Hz, 2H); [α]_D_ = + 67.5° (1 g / 100 mL in CH_2_Cl_2_); *ee* = 96% (*R*).

#### 3.1.2. Synthesis of (*R*)-2,2’-bis(2,2’-bithiophen-5-yl)-1,1’-binaphthalene, (*R*)-Naph_2_T_4_

A mixture of (*R*)-(-)-2,2’-dibromo-1,1’-binaphthalene (1 eq., 182 mg, 0.44 mmol), 2-([2,2’-bithiophen]-5-yl)-4,4,5,5-tetramethyl-1,3,2-dioxaborolane (2.5 eq., 323 mg, 1.1 mmol), Pd(PPh_3_)_4_ (0.1 eq., 51 mg, 0.044 mmol) and Na_2_CO_3_ (3.5 eq., 164 mg, 1.55 mmol) in a degassed mixture of dry toluene (18 mL), ethanol (6 mL) and water (6 mL) was refluxed under argon atmosphere for 24 hours. Then, the reaction was cooled at room temperature and AcOEt was added; the organic phase was washed with water, dried over MgSO_4_, filtered, and the solvent was removed under reduced pressure. The residue was purified by column chromatography (*n*-hexane:CH_2_Cl_2_ = 9:1) to afford the desired product as a yellow solid.

Yield: 67 mg (26%); ^1^H-NMR (CDCl_3_, 400 MHz): δ = 8.04 (2H, d, *J* = 11.6 Hz), 7.93 (2H, d, *J* = 10.8 Hz), 7.89 (2H, d, *J* = 11.2 Hz), 7.46 (2H, m), 7.26–7.21 (4H, m), 7.12 (2H, dd, *J* = 4.0 Hz) 6.92 (2H, broad s) 6.91 (2H, broad s), 6.79 (2H, d, *J* = 5.2 Hz), 6.58 (2H, d, *J* = 5.2 Hz);

^13^C NMR (CDCl_3_, 100 MHz) δ = 141.6, 137.8, 137.4, 134.0, 133.0, 132.8, 132.3, 128.9, 128.0, 127.6, 127.1, 127, 126.9, 126.6, 126.2, 124.1, 123.5, 123.3; [α]_D_ = +206° (0.1 g/100 mL in CH_2_Cl_2_). Optical purity was 99.2%, evaluated by HPLC on a Daicel Chiralpak OD-H column, eluent: 98:2 Hex/IPA; 0.8 mL/min flow rate, detection: 300 nm, *t*_1_ 9.03 min (minor), *t*_2_ 10.70 min (major). M.S. (ESI): 582 (100%), 584 (42%).

#### 3.1.3. Synthesis of (*S*)-2,2’-bis(2,2’-bithiophen-5-yl)-1,1’-binaphthalene, (*S*)-Naph_2_T_4_

The same procedure was carried out starting from (*S*)-(−)-2,2’-dibromide-1,1’-binaphthalene, affording (*S*)-BN_2_T_4_ in 27% yield; [α]_D_ = −206° (0.1 g/100 mL in CH_2_Cl_2_). The optical purity was 97.2%, evaluated by HPLC on a Daicel Chiralpak OD-H column, eluent: 98:2 Hex/IPA; 0.8 mL/min flow rate, detection: 220 nm; *t*_1_ 9.38 min (major), *t*_2_ 11.77 min (minor).

### 3.2. Computational Structural Study

Preliminary energy structures were obtained by MonteCarlo conformational analysis performed with Molecular Mechanics calculations using the OPLS_2005 force field [33] of the Macromodel [34] package in the Schrödinger suite [35], since many conformers are possible due to the mobility of the thiophene pendant groups. In this way, the best arrangement for the different substituents around a binaphthyl scaffold was obtained (Table 1, cut-off: 10.0 Kcal/mol).

According to force field analysis, in the most stable conformation (#1), the two thiophene rings are oriented in a -*trans* arrangement. For this reason, conformation #1 has been employed as the initial structure to calculate the racemization barrier by the Parameterization Method 6 (PM6) semi-empirical method [36] and by the DFT approach. The transition states responsible for the interconversion of the two enantiomers (S) ⮀ (R), were fully optimized to the relative genuine TSs (each with only one imaginary frequency), using B3LYP [37] functional with different basis sets available in the Gaussian G09 rev E.01 package [38]. Results are reported in Table 4. 

### 3.3. Electrochemistry

Voltammetric characterizations, potentiodynamic electrodepositions and voltammetric enantiodiscrimination tests have been carried out using an AutoLab PGStat potentiostat (Eco-Chemie, Utrecht, The Netherlands) controlled by a PC with dedicated GPES software (provided by the same manufacturer) and a classical three-electrode glass minicell (working volume: 2 cm^3^). The latter includes a glassy carbon GC disk working electrode (polished when necessary with synthetic diamond powder (Aldrich, St. Louis, MO, USA) on wet DP (Diamond Pad)-NAP cloth (Struers, Cleveland, OH, USA)), a platinum counter electrode and as reference electrode, a saturated aqueous calomel electrode (SCE) inserted in a compartment with the working medium ending in a porous frit to avoid contamination of the working solution by water and KCl traces.

For the magnetoelectrochemistry experiments, a cuvette as a working cell including the following was used:

- as working electrode, an ITO one, bare or modified with either oligo-BNT_2_T_4_ film antipode (by a single electrodeposition cycle in CH_3_CN +TBAPF_6_ medium);
- a Pt counter electrode
- an aqueous saturated calomel electrode (SCE) inserted in a compartment with porous frit as above described as a reference electrode.

The aqueous working solution contained the achiral redox couple K_3_[Fe(CN)_6_] + K_4_[Fe(CN)_6_] (each of them 2.5 mM) with 400 mM KCl as the supporting electrolyte.

A magnetic field perpendicular to the electrode surface was obtained by placing a permanent magnet (nickel-coated NdFeB B88X0 Grade N42 K&J Magnet, Inc., 6353 Gauss) close to the Working Electrode (WE), at a distance of approximately 2.6 mm, considering the global thickness of both ITO-coated glass electrode and cuvette). The magnetic field orientation was changed by mechanically flipping the magnet around its magnetic axis.

### 3.4. AFM Characterization of the Chiral Electrode Surfaces

AFM imaging was performed in air using a Nanoscope Multimode IIId system (Bruker, SantaBarbara, CA, USA) operating in tapping-mode. AFM images were collected using the rms amplitude of the cantilever as the feedback signal for the vertical sample position. The rms free amplitude of the cantilever was approximately 15 nm and the relative set-point was above 95% of the free amplitude. Rectangular silicon probes with a nominal spring constant around 2.5 N/m (NT-MDT, Russia) and cantilever length of 120 μm were used. The cantilever resonance frequency was about 130 kHz. Images were recorded at a 1 Hz line rate and a resolution of 512 × 512 pixels per image was chosen. AFM images were subject to a line-by-line subtraction of linear background to eliminate sample tilt from the images and correct for stepwise changes between individual scan lines.

## 4. Conclusions

The new inherently chiral monomer **Naph_2_T_4_** (based on a biaromatic rather than biheteroaromatic atropisomeric core) can be advantageously obtained in both enantiopure forms without the need for semipreparative HPLC separation steps via a two-step synthetic route hinging on 1,1′-binaphthyl-2,2′-diamine as a chiral intermediate, which is commercially available in both enantiopure forms at a reasonable price. Comparative theoretical calculations were performed on **Naph_2_T_4_, (N-Me-IND)_2_T_4_** and **BT_2_T_4_** to calculate their racemization barriers, some relevant structural parameters, orbital energies and topologies. The results demonstrate that these monomers constitute a modular series differing in shape and electronic properties. Notably a relevant parameter is the dihedral angle, which is surprisingly very narrow in the case of the new binaphthyl-based monomer.

The antipodes of the new inherently chiral monomer can be easily electrooligomerized, yielding inherently chiral electrode surfaces that perform very well in both CV enantiodiscrimination tests with pharmaceutically interesting molecules where they showed huge affinity for small chiral probes like DOPA and tryptophan, which might be linked to the oligomer nature in connection with the monomer dihedral angle. Remarkable results were also obtained in magnetoelectrochemistry experiments.

In conclusion, a new attractive term has been added to our growing palette of inherently chiral selectors for enantioselective electrochemistry.

## Figures and Tables

**Table 1:** A comparison of torsional energy barrier calculations for different atropisomeric scaffolds, performed by different computational strategies. ^a^ Single point energy values. ^b^ Difference between the energy of the TS and the energy of the fundamental state.

	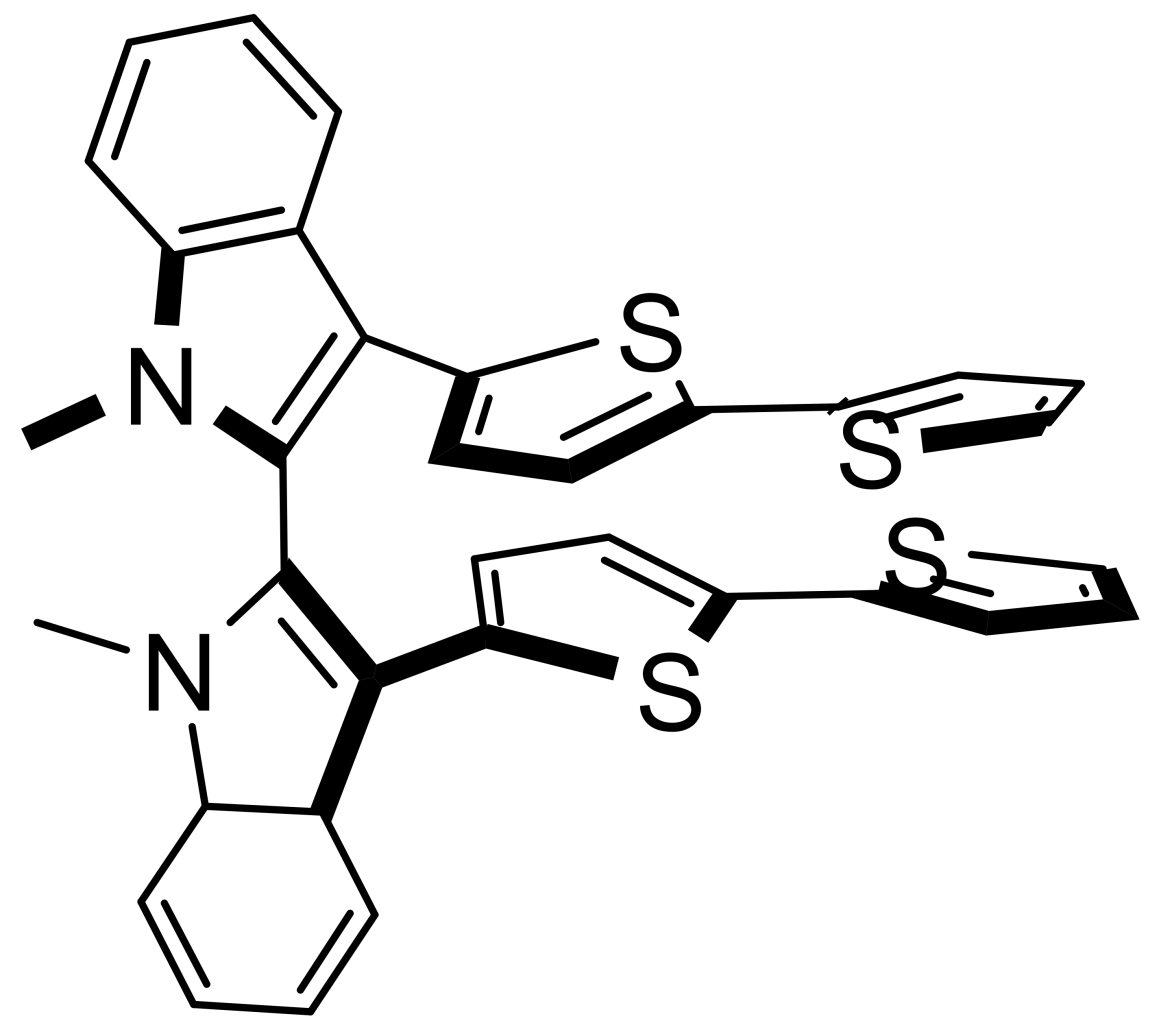 (*S*)-(N-Me-IND)_2_T_4_		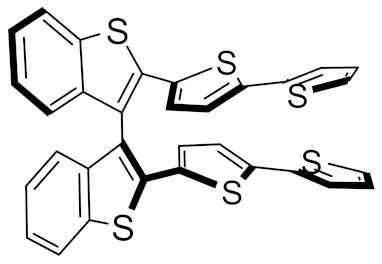 (*S*)-BT_2_T_4_		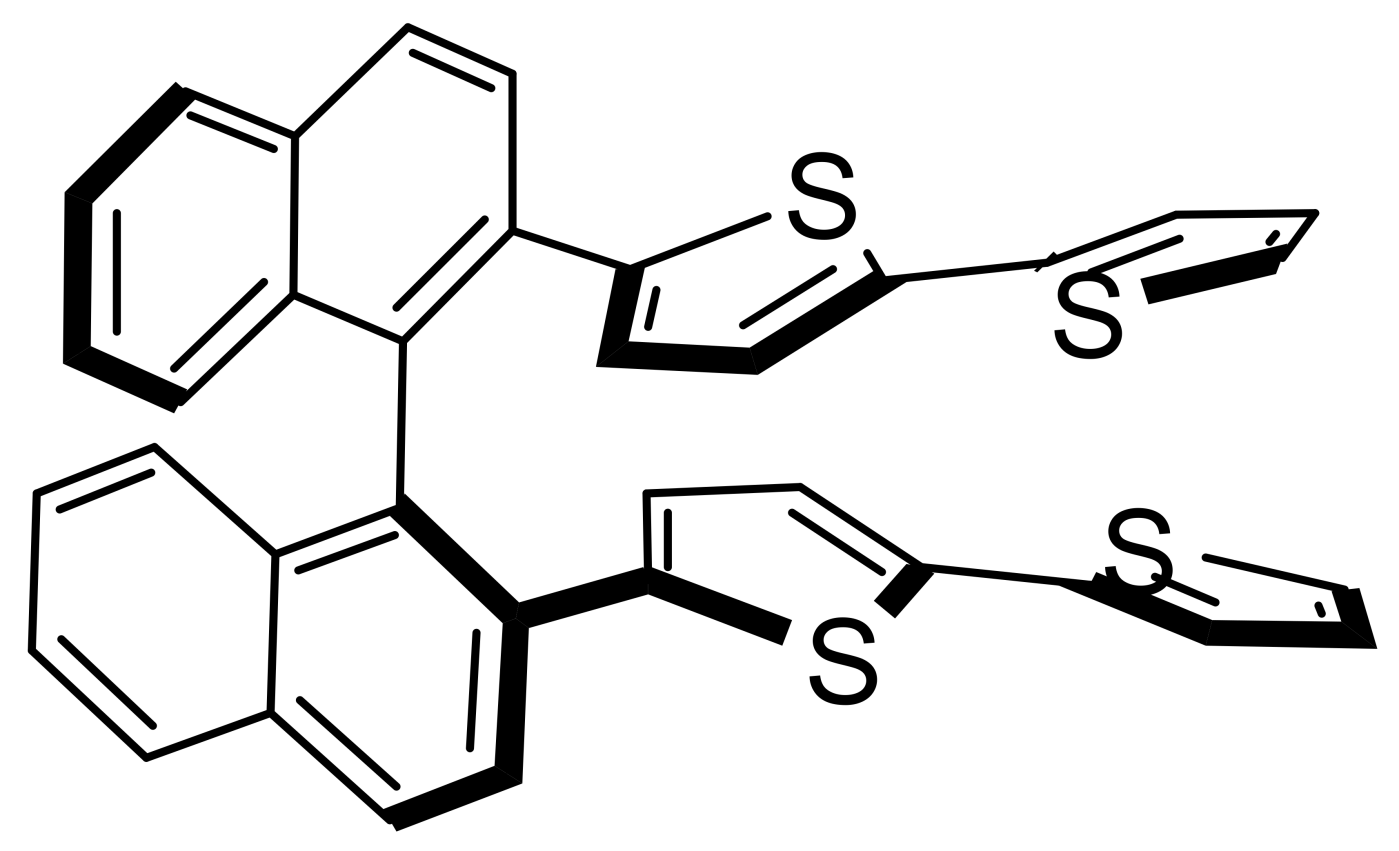 (*S*)-Naph_2_T_4_	
Level of Theory	TS1 ΔΔG^≠^ (ΔΔE^≠^) ^b^ (kcal/mol)	TS2 ΔΔG^≠^ (ΔΔE^≠^) ^b^ (kcal/mol)	TS1 ΔΔG^≠^ (ΔΔE^≠^) (kcal/mol)	TS2 ΔΔG^≠^ (ΔΔE^≠^) (kcal/mol)	TS1 ΔΔG^≠^ (ΔΔE^≠^) (kcal/mol)	TS2 ΔΔG^≠^ (ΔΔE^≠^) (kcal/mol)
PM6	24.77 (20.75)	28.56 (24.06)	25.32 (21.25)	25.95 (22.42)	36.17 (33.30)	35.22 (32.92)
B3LYP/6-31G(d,p)	30.14 (26.90)	36.46 (33.38)	38.55 (34.76)	39.78 (35.94)	46.15 (43.95)	46.64 (44.76)
B3LYP-D3/6-31G(d,p)	28.77 (26.32)	30.90 (27.58)	35.07 (33.78)	34.53 (32.53)	45.49 (45.43)	46.61 (46.41)
B3LYP/TZVP	30.68 (27.19)	36.49 (33.47)	39.04 (35.50)	40.42 (36.32)	46.09 (43.95)	47.02 (44.76)
B3LYP/6 − 311G + (3df,3pd) // B3LYP/6-31G(d,p) ^a^	- (27.38)	- (33.17)	- (35.42)	- (36.63)	- (43.77)	- (44.54)

**Table 2:** Rms surface roughness values (on 500 × 500 nm^2^ areas) of the two films **(*R*)-Naph_2_T_4_** CH_2_Cl_2_ and **(R)-Naph_2_T_4_** CH_3_CN, as obtained by atomic force microscopy (AFM) imaging. SD: standard deviation.

Sample	Rms Surface Roughness (nm)
**oligo-(*R*)-Naph_2_T_4_** CH_2_Cl_2_	6.1 ± 2.8 (mean ± SD; *n* = 30)
**oligo-(*R*)-Naph_2_T_4_** CH_3_CN	14.5 ± 6.7 (mean ± SD; *n* = 30)

**Table 3:** Testing potential differences (Δ*E*) observed for the enantiomers of FcA and DOPA in aqueous solution with 0.05 M HCl as the supporting electrolyte on GC modified with enantiopure oligo-**Naph_2_T_4_,** oligo-**BT_2_T_4_**, and oligo-**(N-Me-IND)_2_T_4_** films (with respective monomer dihedral angles).

Monomer	Dihedral Angle	Δ*E* FcA/mV	Δ*E* DOPA/mV
**Naph_2_T_4_**	78	70	450
**BT_2_T_4_**	101	45	100
**(N-Me-IND)_2_T_4_**	117.61	270	200

**Table 4:** Dihedral angles of **Naph_2_T_4_** obtained by force field analysis.

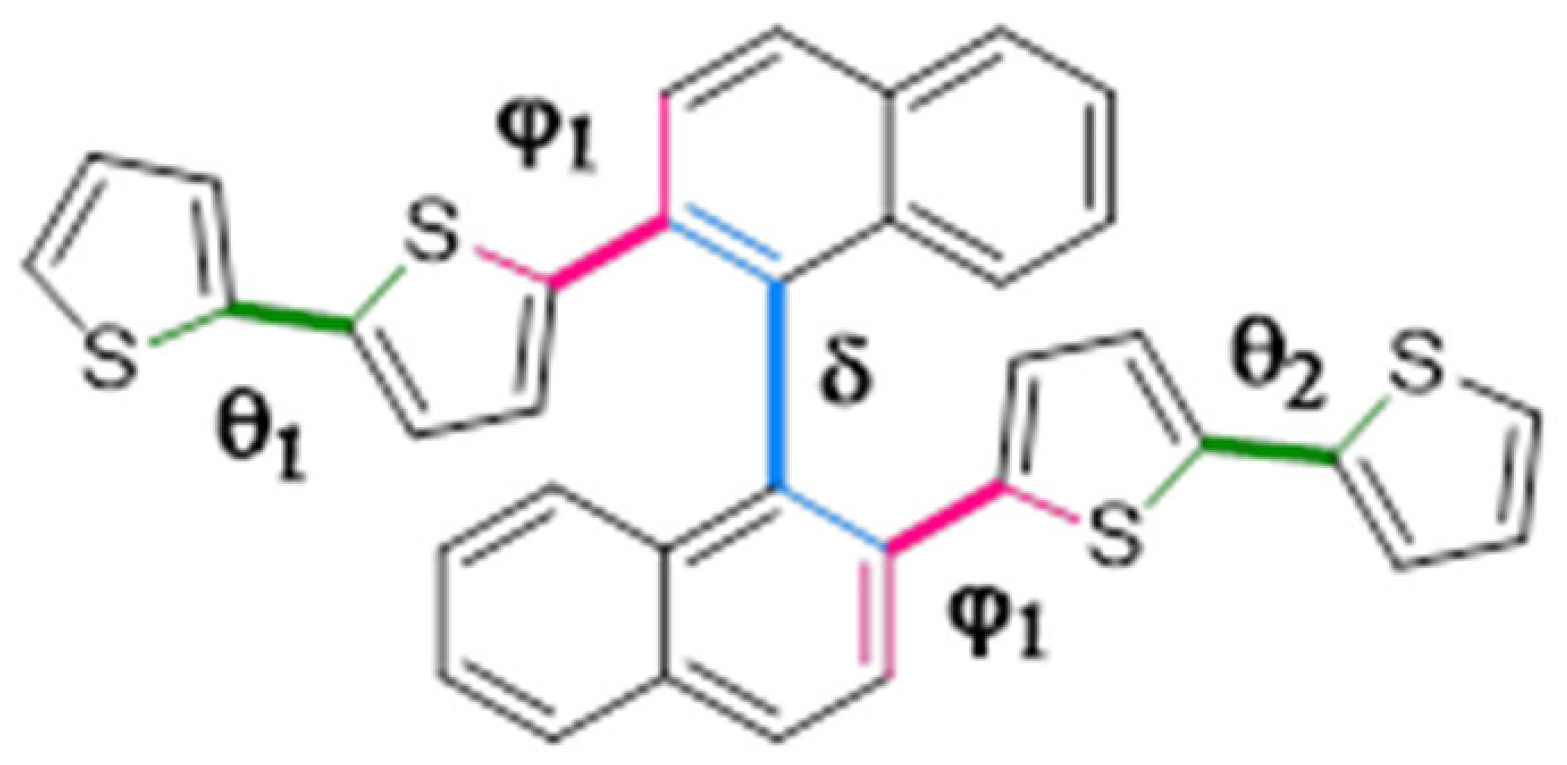						
#	Δ*E* * (kcal/mol)	δ (Degree)	φ_1_ (Degree)	φ_2_ (Degree)	θ_1_ (Degree)	θ_2_ (Degree)
1	0	−75.38	−50.98	−50.66	169.60	170.06
2	3.17	−75.69	−50.13	−52.68	29.54	149.99
3	3.40	−76.82	−51.29	−51.82	−33.15	172.30
4	6.54	75.86	52.00	50.05	36.64	−36.37
5	6.63	78.68	51.31	−112.95	179.78	167.70
6	7.73	−78.28	−52.43	−52.40	−32.50	−32.39
7	9.08	78.22	51.81	−113.73	36.58	168.14
8	9.74	−78.46	113.13	−51.04	35.59	−176.53
9	9.97	78.91	51.60	−111.99	−30.66	167.25

* calculated with OPLS_2005 force field.

## Supplementary Materials

SI 1. Computational details; SI 2. LDI spectra of cyclic dimer and trimer in the Soxhlet THF-extracts of the FeCl3 oligomerization reaction mixture; SI 3. Enantiosdiscrimination test with benchmark ferrocenyl chiral probe on enantiopure oligo-(R)-BTN2T4; SI 4. 1H NMR and 13C NMR spectra of Naph2T4; SI 5. HPLC chromatograms of (R)- and (S)- Naph2T4.
