# Supplementary material for: Effective Enantiodiscrimination in Electroanalysis Based on a New Inherently Chiral 1,1′-Binaphthyl Selector Directly Synthesizable in Enantiopure Form
Source: Molecules. 2020 May 6;25(9):2175. doi: 10.3390/molecules25092175 (PMC7249101; doi:10.3390/molecules25092175)
Supplement: Supplementary file 1 [file molecules-25-02175-s001.pdf]

molecules
MDPI

Supplementary Information

Effective Enantiodiscrimination in Electroanalysis Based on a New Inherently Chiral 1,1′-binaphthyl Selector Directly Synthesizable in Enantiopure Form

Giorgia Bonetti [1], Serena Arnaboldi [2], Sara Grecchi [2], Giulio Appoloni [1], Elisabetta Massolo [2], Sergio Rossi [2], Rocco Martinazzo [2], Francesco Orsini [3], Patrizia R. Mussini [2] and Tiziana Benincori [1,*]

[1] Dipartimento di Scienza ed Alta Tecnologia, Università degli Studi dell'Insubria, Como, 22100 Italy; gbonetti@uninsubria.it (G.B.); appogiulio@yahoo.it (G.A.)
[2] Dipartimento di Chimica, Università degli Studi di Milano, 20133 Milano, Italy; serena.arnaboldi@unimi.it (S.A.); sara.grecchi@unimi.it (S.G.); elisabetta.massolo@unimi.it (E.M.); sergio.rossi@unimi.it (S.R.); rocco.martinazzo@unimi.it (R.M.); patrizia.mussini@unimi.it (P.R.M.)
[3] Dipartimento di Fisica, Università degli Studi di Milano, 20133, Milano, Italy, francesco.orsini@unimi.it
* Correspondence: tiziana.benincori@uninsubria.it; Tel.: +39-031-2386615

SI 1. Computational details
SI 2. LDI spectra of cyclic dimer and trimer in the Soxhlet THF-extracts of the $FeCl_3$ oligomerization reaction mixture
SI 3. Enantiosdiscrimination test with benchmark ferrocenyl chiral probe on enantiopure oligo-(*R*)-BTN_2T_4
SI 4. ^{1}H NMR and ^{13}C NMR spectra of $Naph_2T_4$
SI 5. HPLC chromatograms of (*R*)- and (*S*)- $Naph_2T_4$

SI. 1 Computational Details

$Naph_2T_4$ - Global minimum (B3LYP/6-311G+(3df,3pd) // B3LYP/6-31G(d,p)

C	1.51700	-1.11800	1.67200
C	0.86000	-1.55500	0.49200
C	-0.14400	-0.74300	-0.21000
C	-0.03100	0.73700	-0.19100
C	-1.09400	1.67300	-0.56800
C	-1.99700	1.45000	-1.63600
C	-2.99600	2.35200	-1.94900
C	-3.15600	3.53500	-1.20000
C	-2.23000	3.84000	-0.22700
C	-1.15300	2.96300	0.05700
C	-0.05100	3.40100	0.83900
C	1.11500	2.68800	0.78400
C	1.18200	1.39200	0.18900
C	-1.18100	-1.52300	-0.81000
C	-0.88100	-2.85200	-1.23600
C	0.28700	-3.48600	-0.91000
C	1.12500	-2.89900	0.07400
C	2.13500	-3.66100	0.71600
C	2.79800	-3.17100	1.81800
C	2.44500	-1.90300	2.32400
H	1.25400	-0.15700	2.09500
H	2.90200	-1.54000	3.23900
H	3.55300	-3.77000	2.31900
H	2.33500	-4.66300	0.34800
H	0.50000	-4.48700	-1.27400
H	-1.64600	-3.38100	-1.79500
H	-1.87000	0.57900	-2.25800
H	-3.65200	2.15000	-2.79000
H	-3.96300	4.22400	-1.42900
H	-2.27200	4.79100	0.29800
H	-0.09500	4.36300	1.34000
H	2.03000	3.12100	1.17600
C	2.51700	0.88000	-0.13300
C	2.90200	0.16800	-1.25100
C	5.01800	0.50500	-0.30900
H	2.19000	-0.17000	-1.99400
C	-2.61500	-1.20000	-0.87800

C	-3.53300	-1.50800	-1.86100
C	-5.01200	-0.70400	-0.23600
C	-4.87400	-1.21600	-1.51000
H	-3.24100	-1.89800	-2.83000
H	-5.71400	-1.35600	-2.18100
C	-6.21600	-0.29200	0.45500
C	-6.35100	0.56000	1.53100
C	-7.70000	0.73700	1.95000
H	-5.50600	1.06200	1.98800
C	-8.59100	0.02400	1.19600
H	-7.99400	1.37700	2.77400
H	-9.66700	-0.02000	1.28800
S	3.93600	1.29300	0.82100
S	-3.44800	-0.60000	0.54500
S	-7.79100	-0.89600	-0.03900
C	6.44800	0.49600	-0.08500
C	7.20400	1.29500	0.74700
C	8.90200	-0.01800	-0.15100
H	6.77200	2.08400	1.35300
C	8.59600	1.00100	0.70900
H	9.87100	-0.43800	-0.37900
H	9.33900	1.53300	1.29100
S	7.48300	-0.65100	-0.92300
C	4.29600	-0.04700	-1.34800
H	4.76500	-0.56400	-2.17800

$Naph_2T_4$ - TS1 (B3LYP/6-311G+(3df,3pd) // B3LYP/6-31G(d,p))

C	1.51700	-1.11800	1.67200
C	0.86000	-1.55500	0.49200
C	-0.14400	-0.74300	-0.21000
C	-0.03100	0.73700	-0.19100
C	-1.09400	1.67300	-0.56800
C	-1.99700	1.45000	-1.63600
C	-2.99600	2.35200	-1.94900
C	-3.15600	3.53500	-1.20000
C	-2.23000	3.84000	-0.22700
C	-1.15300	2.96300	0.05700
C	-0.05100	3.40100	0.83900
C	1.11500	2.68800	0.78400
C	1.18200	1.39200	0.18900

C	-1.18100	-1.52300	-0.81000
C	-0.88100	-2.85200	-1.23600
C	0.28700	-3.48600	-0.91000
C	1.12500	-2.89900	0.07400
C	2.13500	-3.66100	0.71600
C	2.79800	-3.17100	1.81800
C	2.44500	-1.90300	2.32400
H	1.25400	-0.15700	2.09500
H	2.90200	-1.54000	3.23900
H	3.55300	-3.77000	2.31900
H	2.33500	-4.66300	0.34800
H	0.50000	-4.48700	-1.27400
H	-1.64600	-3.38100	-1.79500
H	-1.87000	0.57900	-2.25800
H	-3.65200	2.15000	-2.79000
H	-3.96300	4.22400	-1.42900
H	-2.27200	4.79100	0.29800
H	-0.09500	4.36300	1.34000
H	2.03000	3.12100	1.17600
C	2.51700	0.88000	-0.13300
C	2.90200	0.16800	-1.25100
C	5.01800	0.50500	-0.30900
H	2.19000	-0.17000	-1.99400
C	-2.61500	-1.20000	-0.87800
C	-3.53300	-1.50800	-1.86100
C	-5.01200	-0.70400	-0.23600
C	-4.87400	-1.21600	-1.51000
H	-3.24100	-1.89800	-2.83000
H	-5.71400	-1.35600	-2.18100
C	-6.21600	-0.29200	0.45500
C	-6.35100	0.56000	1.53100
C	-7.70000	0.73700	1.95000
H	-5.50600	1.06200	1.98800
C	-8.59100	0.02400	1.19600
H	-7.99400	1.37700	2.77400
H	-9.66700	-0.02000	1.28800
S	3.93600	1.29300	0.82100
S	-3.44800	-0.60000	0.54500
S	-7.79100	-0.89600	-0.03900
C	6.44800	0.49600	-0.08500
C	7.20400	1.29500	0.74700
C	8.90200	-0.01800	-0.15100

H	6.77200	2.08400	1.35300
C	8.59600	1.00100	0.70900
H	9.87100	-0.43800	-0.37900
H	9.33900	1.53300	1.29100
S	7.48300	-0.65100	-0.92300
C	4.29600	-0.04700	-1.34800
H	4.76500	-0.56400	-2.17800

$Naph_2T_4$ - TS2 (B3LYP/6-311G+(3df,3pd) // B3LYP/6-31G(d,p))

C	4.69900	-0.15100	-1.05600
C	3.44100	-0.68600	-1.44100
C	2.18200	-0.44500	-0.72200
C	2.01800	-0.37100	0.75100
C	3.10200	-0.23700	1.73400
C	4.36000	-0.88800	1.63200
C	5.34700	-0.72900	2.58600
C	5.14100	0.10400	3.70400
C	3.89900	0.66500	3.89500
C	2.84700	0.44900	2.96900
C	1.50700	0.76800	3.31600
C	0.49200	0.20700	2.59200
C	0.72300	-0.45800	1.35200
C	1.06500	-0.25600	-1.58900
C	1.05500	-0.89700	-2.86000
C	2.15200	-1.55400	-3.35000
C	3.40400	-1.35700	-2.70900
C	4.61100	-1.67700	-3.38300
C	5.82600	-1.22900	-2.91500
C	5.85400	-0.40800	-1.77000
H	4.75300	0.51800	-0.21000
H	6.78800	0.04500	-1.45100
H	6.74200	-1.45400	-3.45300
H	4.54700	-2.22800	-4.31700
H	2.11100	-2.08000	-4.30000
H	0.15300	-0.82900	-3.45800
H	4.53800	-1.56800	0.81200
H	6.28300	-1.26800	2.47600
H	5.93200	0.25000	4.43300
H	3.68100	1.23200	4.79700

H	1.30500	1.31500	4.23300
H	-0.52200	0.24400	2.97700
C	-0.06400	0.64600	-1.33900
C	-1.36000	0.55000	-1.80600
H	-1.74600	-0.34600	-2.27800
C	-2.15300	1.69600	-1.55300
C	-1.47600	2.70400	-0.89900
H	-3.20200	1.76700	-1.81800
C	-1.95700	4.00100	-0.47600
C	-1.41300	4.87100	0.44600
C	-3.28000	6.11200	-0.18000
H	-0.51000	4.64600	1.00100
C	-2.16500	6.06800	0.61100
H	-4.02600	6.89100	-0.25100
H	-1.89100	6.86100	1.29800
C	-0.36100	-1.30500	0.84500
C	-0.26100	-2.51100	0.18000
H	0.69000	-2.91700	-0.14400
C	-1.49700	-3.16200	-0.02900
C	-2.58200	-2.47900	0.48900
H	-1.59200	-4.11400	-0.54000
C	-3.98300	-2.84100	0.46600
C	-5.08400	-2.07800	0.79400
H	-5.00800	-1.04600	1.11900
C	-6.32000	-2.76900	0.64800
C	-6.16400	-4.05400	0.20800
H	-7.28600	-2.32300	0.85800
H	-6.92500	-4.79500	0.00800
S	0.18500	2.22300	-0.60300
S	-3.42500	4.68600	-1.15900
S	-2.04600	-0.99700	1.25100
S	-4.49100	-4.45000	-0.02800

SI.2 LDI spectra of cyclic dimer and trimer in the Soxhlet THF-extracts of the $FeCl_3$ oligomerization reaction mixture

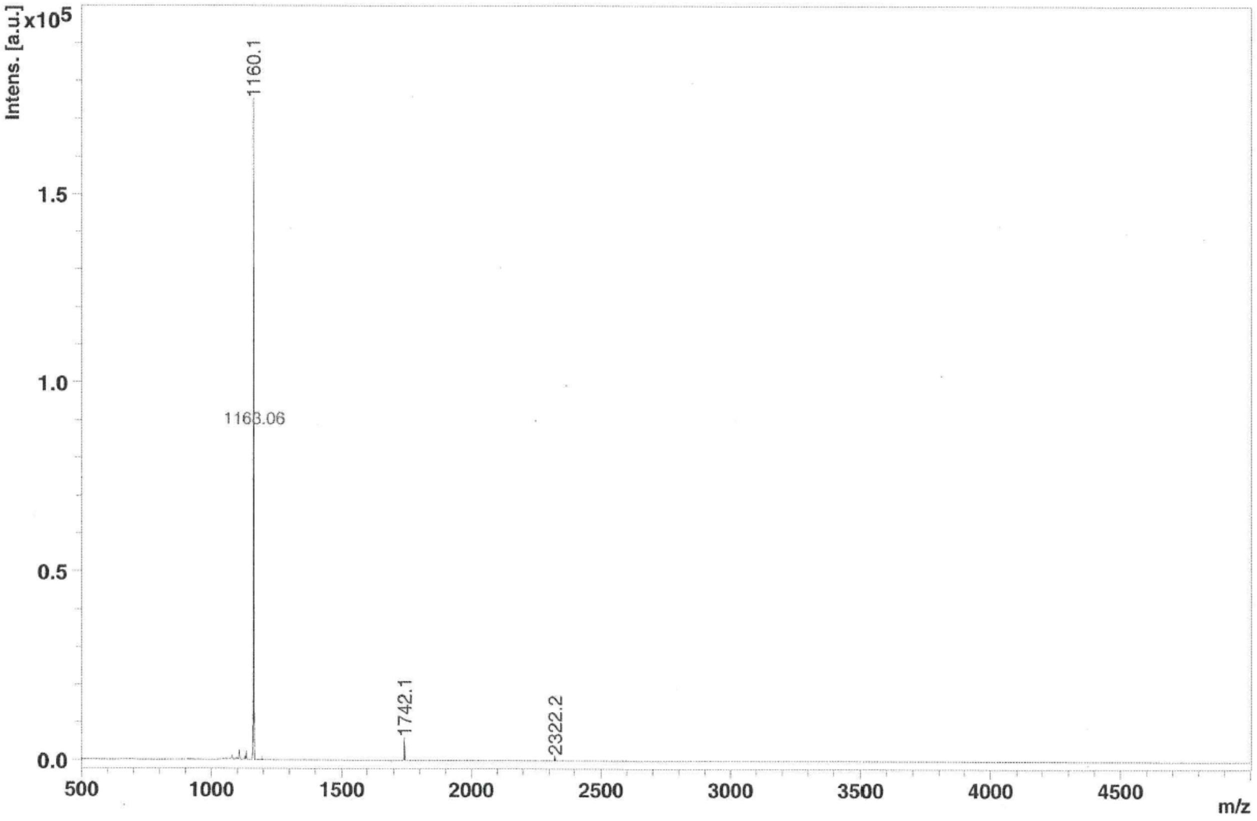

Figure S1. LDI spectrum of the Soxhlet THF extracts.

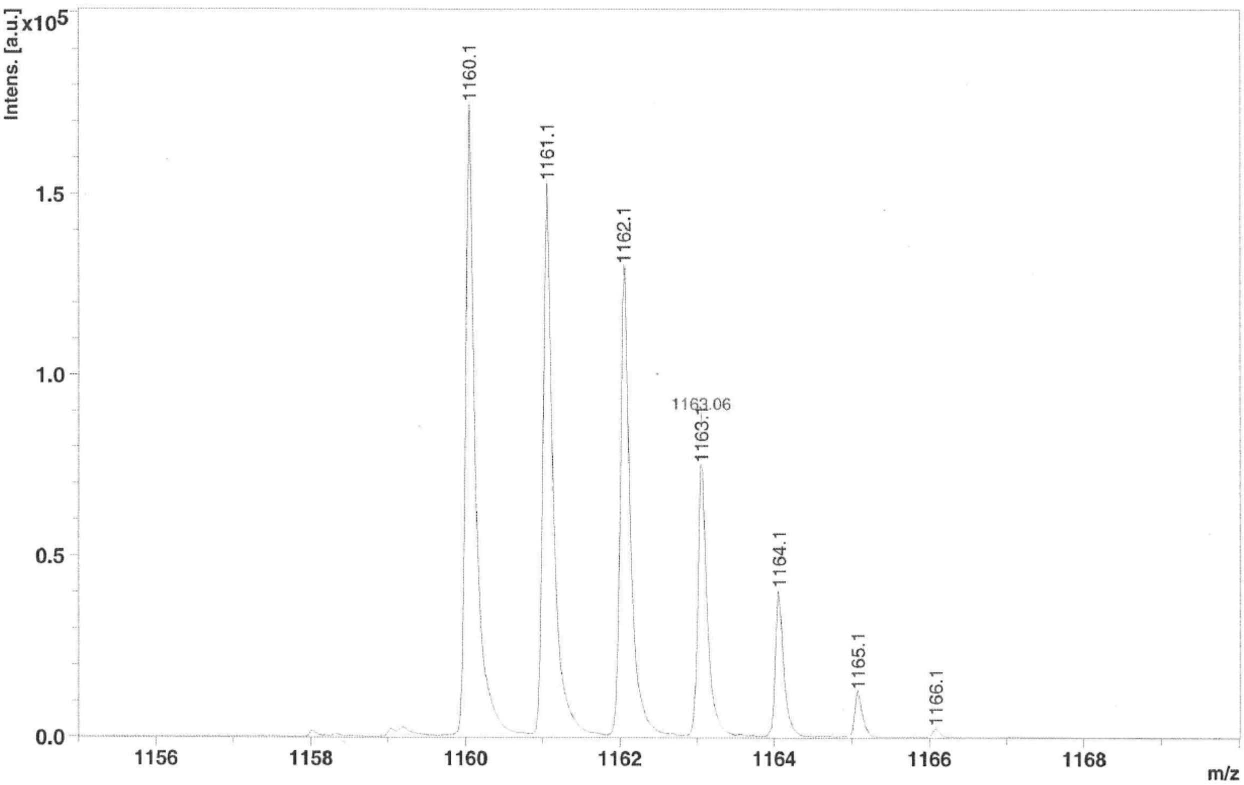

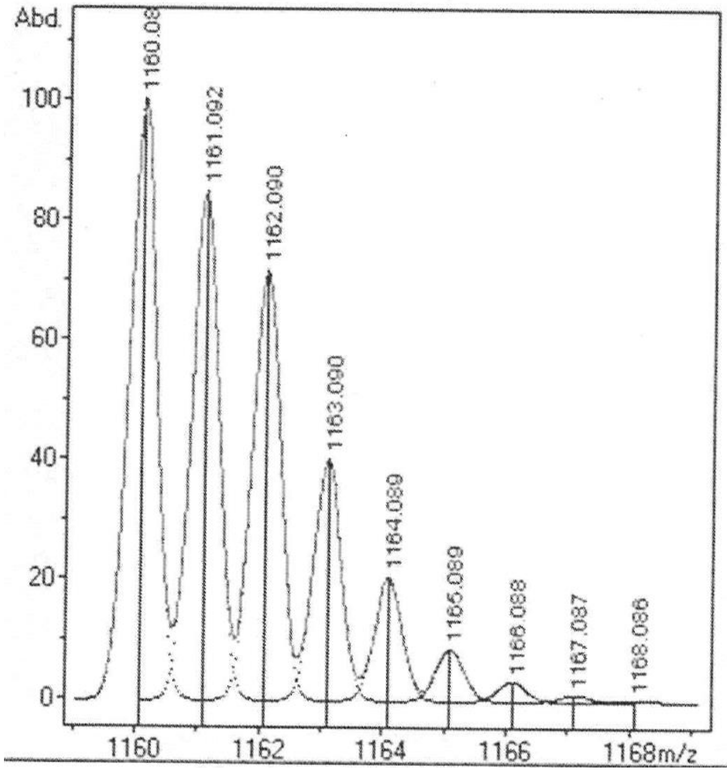

#	m/z	Abundance
1	1160.089018	100.000
2	1161.092089	84.738
3	1162.089936	71.609
4	1163.090430	40.111
5	1164.089056	20.454
6	1165.088688	8.646
7	1166.087627	3.315
8	1167.086955	1.128
9	1168.086050	0.351

Figure S2. Experimental (top) and calculated $C_{72}H_{40}S_8$ (bottom) High Resolution LDI mass spectra in the range m/z = 1160-1170 .

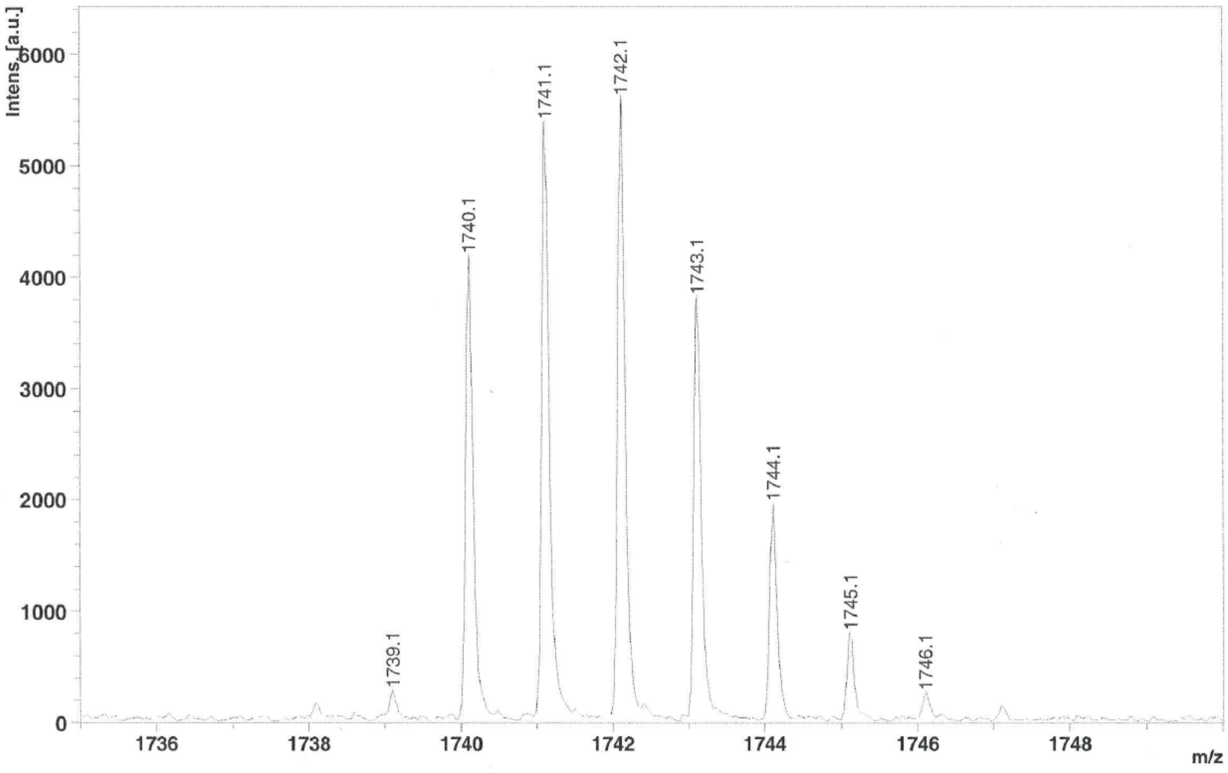

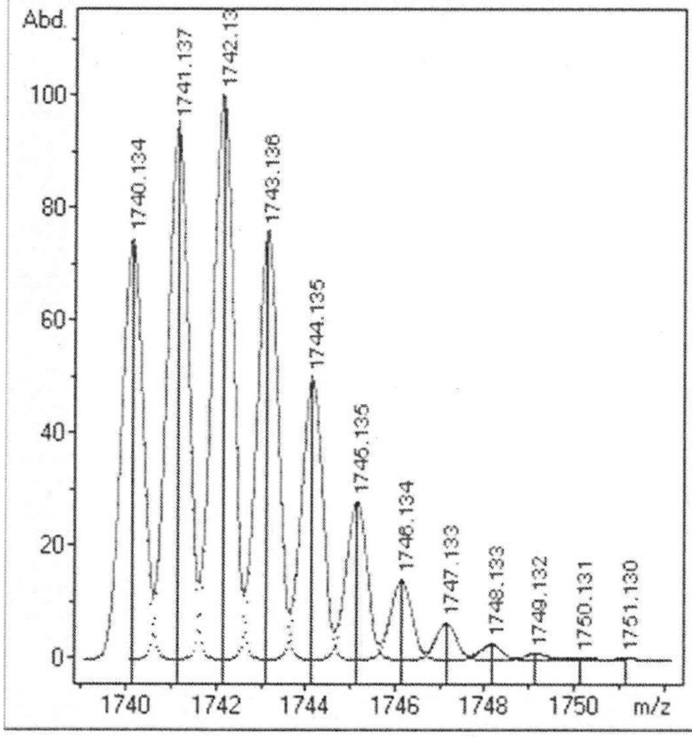

#	m/z	Abundance
1	1740.133802	74.438
2	1741.136872	94.616
3	1742.135767	100.000
4	1743.136073	75.833
5	1744.135257	49.851
6	1745.134842	27.941
7	1746.134054	14.043
8	1747.133385	6.346
9	1748.132584	2.628
10	1749.131821	1.003
11	1750.131016	0.355
12	1751.130229	0.118

Figure S3. Experimental (top) and calculated $C_{108}H_{60}S_{12}$ (bottom) High Resolution LDI mass spectra in the range m/z = 1740-1751.

SI.3. Enantiosdiscrimination test with benchmark ferrocenyl chiral probe on enantiopure Oligo-(*R*)-Naph$_2$T$_4$

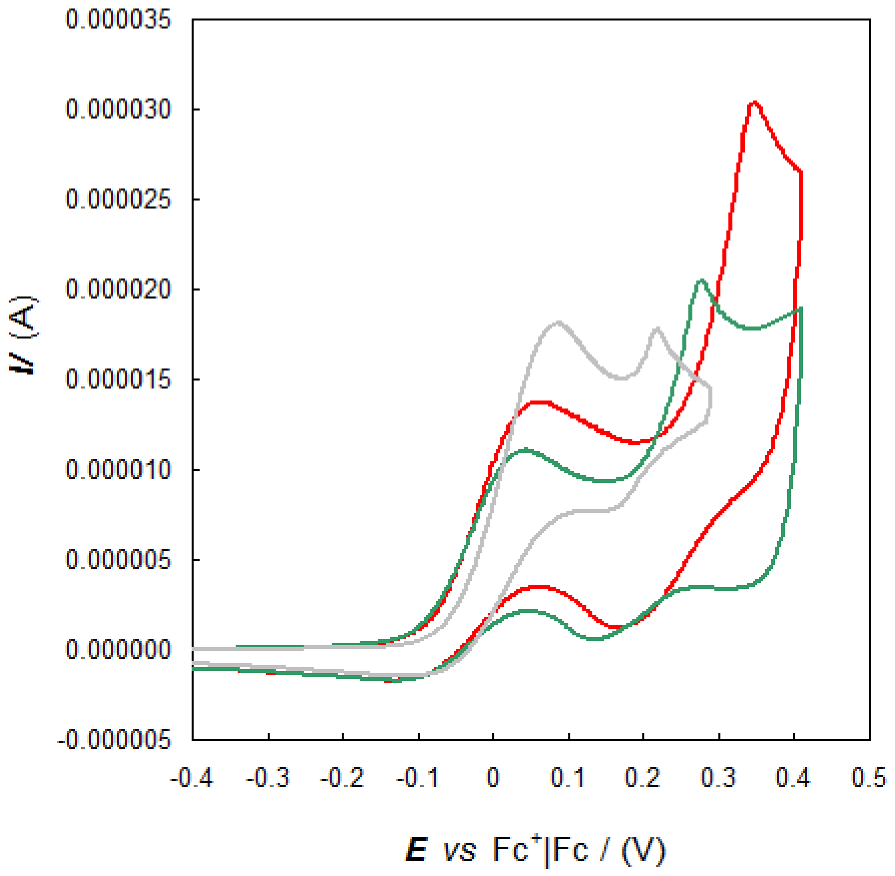

Figure S4. Enantiodiscrimination CV tests with "standard" chiral probe N,N′-dimethyl-1-ferrocenylethylamine, tested in dichloromethane with 0.1M TBAPF$_6$ as supporting electrolyte, on (*S*)- (red curve) or (*R*)- (green curve) oligo-(Naph$_2$T$_4$) films electrodeposited in dichloromethane. As a comparison the CV pattern of achiral ferrocene is also reported (grey curve). The potentials are referred to the formal potential of the ferrocene couple reversible peak in dichloromethane on *bare* GC electrode.

SI 4. ^{1}H NMR and ^{13}C NMR spectra of Naph$_2$T$_4$

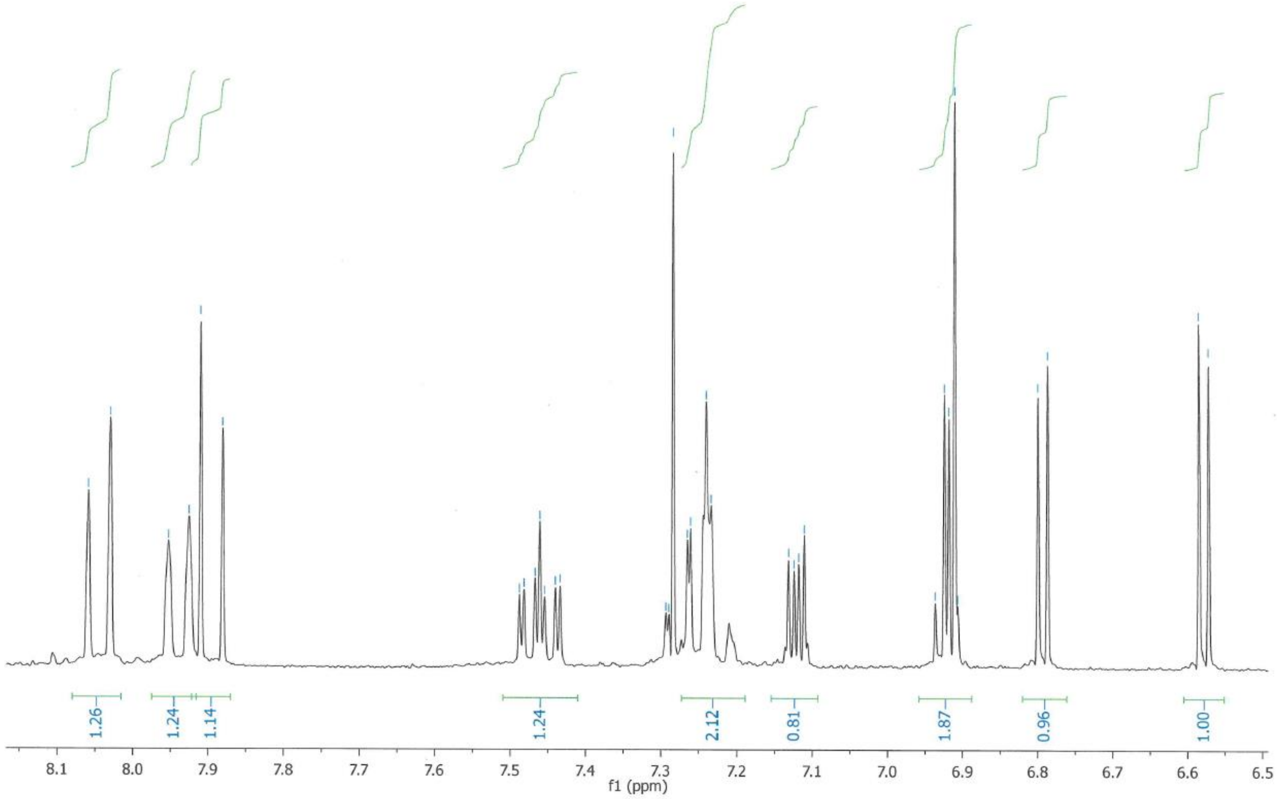

Figure S5. ^{1}H NMR spectrum of Naph$_2$T$_4$ in $CDCl_3$.

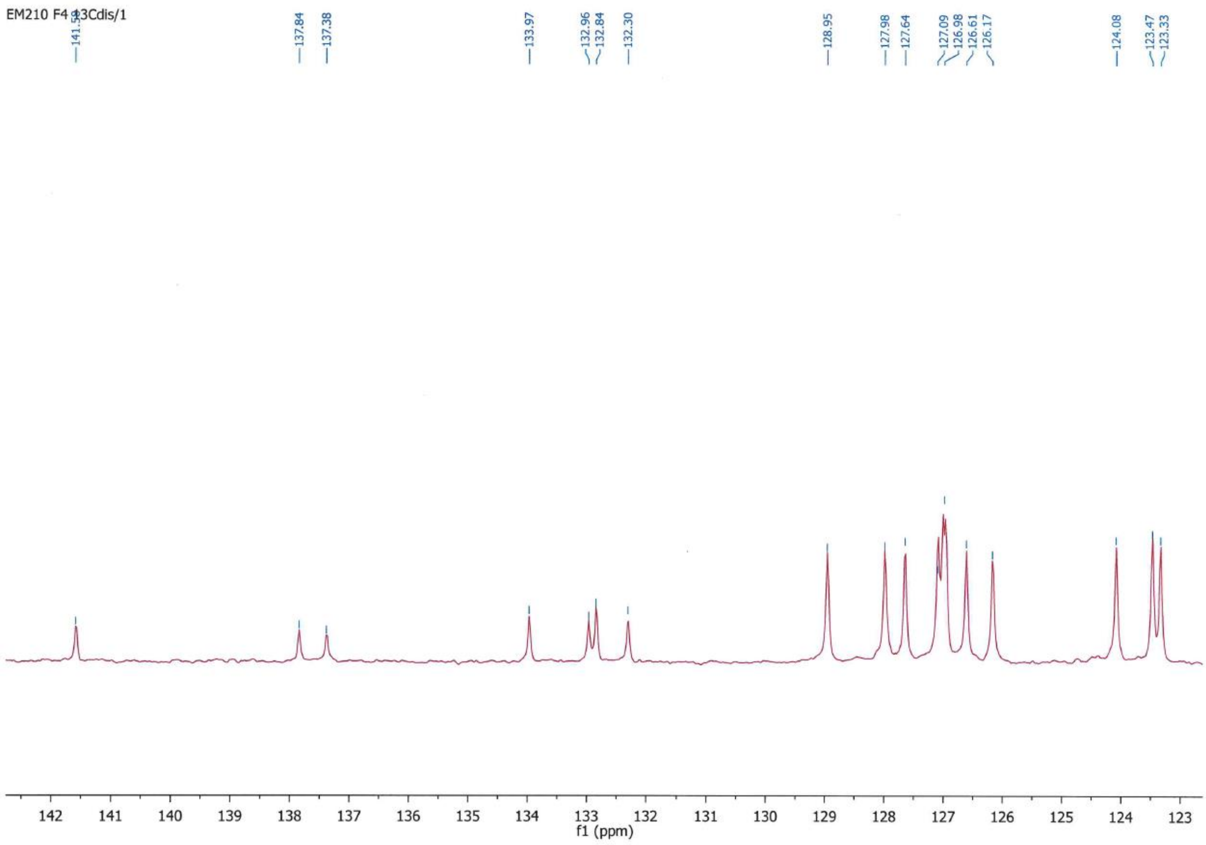

Figure S6. ^{13}C NMR spectrum of $Naph_2T_4$ in $CDCl_3$.

SI 5. HPLC chromatograms of (*R*)- and (*S*)- $Naph_2T_4$

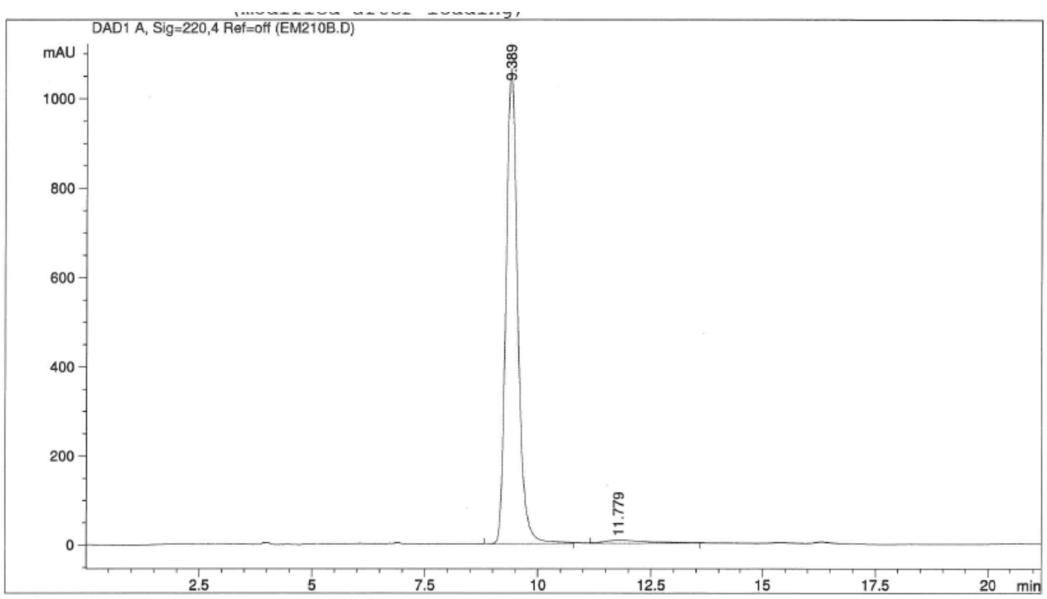

Peak #	RetTime [min]	Type	Width [min]	Area [mAU*s]	Height [mAU]	Area %
1	9.389	VV	0.2800	1.95353e4	1065.13135	97.2171
2	11.779	VV	0.8491	559.20020	7.75615	2.7829
Totals :				2.00945e4	1072.88750	

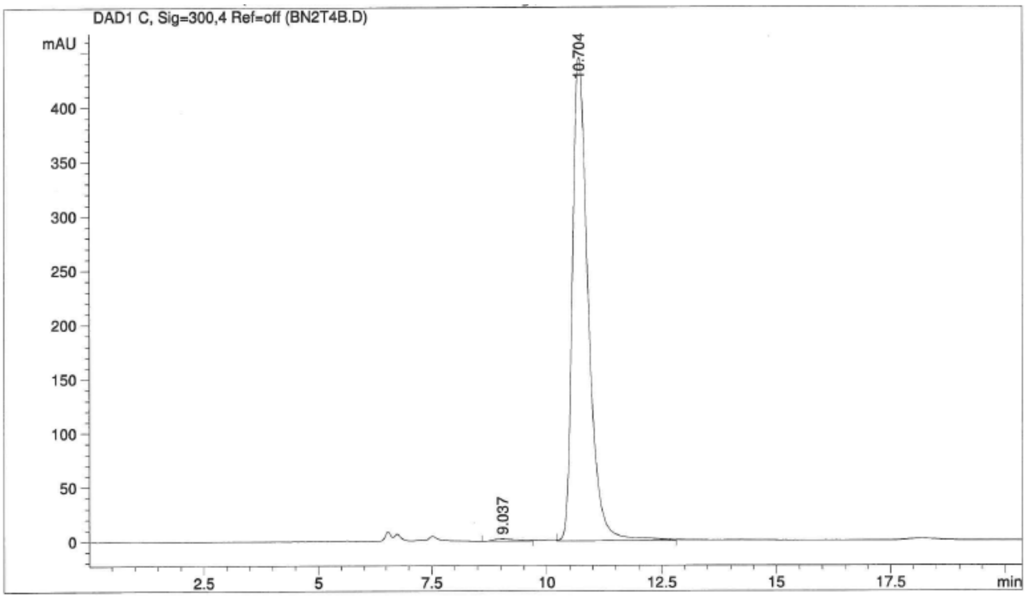

Peak #	RetTime [min]	Type	Width [min]	Area [mAU*s]	Height [mAU]	Area %
1	9.037	PV	0.4825	83.09236	2.06050	0.7714
2	10.704	VV	0.3639	1.06883e4	445.56134	99.2286
Totals :				1.07713e4	447.62184	

Figure S7. HPLC chromatograms of (*S*)-(top) and (*R*)-Naph$_2$T$_4$ (bottom).
